# Degeneracy in neuronal bifurcation landscapes: Equivalent bifurcation sequences across distinct parameter sets

**DOI:** 10.1371/journal.pcbi.1014893

**Published:** 2026-10-30

**Authors:** Gianmarco Cafaro, Christophe Bernard, Meysam Hashemi, Damien Depannemaecker

**Affiliations:** Aix Marseille Univ, INSERM, INS, Inst Neurosci Syst, Marseille, France; Medical University Hannover, GERMANY

## Abstract

Degeneracy, the capacity of structurally distinct systems to achieve similar functions, is a fundamental property of living organisms, enabling adaptability and resilience. Neurons can maintain stable activity patterns despite substantial variability in ion channel expression, highlighting how different intrinsic configurations can produce equivalent electrophysiological behaviors. Previous studies have shown that neuronal activity emerges from the interaction between intrinsic membrane properties and ionic concentration dynamics. However, how degeneracy is organized within the dynamical landscape of neurons and how different degenerate configurations respond to external perturbations remain poorly understood. Here, we investigate how distinct biophysical parameter sets generate equivalent bifurcation sequences and how their resilience changes under ionic modulation. Using a minimal neuron model with three variable conductances, we systematically explore how variations in extracellular potassium concentration, representing physiological and pathological conditions, reshape neuronal dynamics. We show that distinct conductance configurations occupy structured regions of parameter space that support the same bifurcation pathways, leading to equivalent activity regimes such as bursting, seizure-like activity, and depolarization block. However, these degenerate configurations exhibit different sensitivities to extracellular perturbations, revealing that functional equivalence under fixed conditions does not imply equivalent robustness. To quantify this effect, we introduce resilience as a measure of the threshold at which a given conductance configuration transitions into a specific activity regime under external ionic changes. Our results demonstrate that degeneracy extends beyond the coexistence of multiple solutions: degenerate configurations form structured landscapes with distinct trajectories under environmental modulation. This framework provides a quantitative approach to investigate how intrinsic variability influences neuronal vulnerability and resilience across different dynamical states.

## Author summary

Biophysical models expressed through differential equations can reproduce the dynamics of biological systems with varying levels of detail. By changing model parameters, simulations can capture the natural variability that allows biological systems to achieve the same behavior through different mechanisms. This property, called degeneracy, underlies the robustness of living systems to internal and external perturbations. In this work, we identify distinct behaviors in single-neuron models and link them to electrophysiological patterns observed under elevated extracellular potassium during epileptic events. These patterns correspond to specific classes of spontaneous bursting activity within the framework of nonlinear dynamics. We show that different combinations of conductance parameters, at a fixed extracellular potassium level, can generate the same class of patterns, defining degeneracy groups. Finally, we assess the robustness of these groups by analyzing how they respond to changes in extracellular potassium. Our findings provide a basis for studying variability and resilience in the dynamics of more complex neuronal systems.

## 1. Introduction

Degeneracy—the ability of structurally distinct systems to produce similar functional outcomes—is a pervasive feature of biological systems [1]. In the nervous system, this property enables neurons to maintain robust activity despite substantial variability in ion channel expression, synaptic connectivity, and membrane properties [2,3]. Seminal studies by Eve Marder and colleagues demonstrated that individual neurons can compensate for perturbations in internal parameters—such as ionic conductances or channel densities—while preserving stable firing patterns [4,5]. This flexibility is thought to underlie the resilience of neural circuits to genetic, developmental, and environmental variability. Likewise, the initial configuration of a neuron’s parameters determines how it will respond to external modulations such as temperature, pH, or ionic concentrations [6].

While degeneracy in steady-state or repetitive spiking activity is well characterized, far less is known about how it manifests in the transitions between activity regimes—so-called bifurcations—that are central to both normal and pathological dynamics. In epilepsy and related disorders, neurons often display abrupt shifts in excitability, transitioning between silence, bursting, seizure-like discharges, and depolarization block [7,8]. These transitions can be mathematically described as bifurcations in nonlinear dynamical systems, where changes in a control parameter (such as extracellular potassium) lead to qualitative changes in neuronal behavior [9,10].

Epileptic activity has been observed across a wide range of species and brain structures [11–13], and may arise from diverse etiologies including genetic, traumatic, or metabolic origins [14–16]. Despite this heterogeneity, electrophysiological signatures such as rhythmic bursting or sustained ictal activity are remarkably conserved. Canonical dynamical models reproduce these activity patterns using low-dimensional systems of differential equations that capture the onset and offset bifurcations defining epileptiform transitions [7,17]. These phenomenological models can be linked to biophysically grounded descriptions, which incorporate specific ionic currents and concentration dynamics [9,18,19].

A central question is whether different biophysical implementations—distinct combinations of ionic conductances—can give rise to the same sequence of bifurcations under changing environmental conditions. In particular, how does degeneracy manifest when neurons are exposed to elevated extracellular potassium concentration, a hallmark of intense neuronal activity and epileptic seizures [20–23]?

To address this, we employ a simplified single-compartment neuron model with three tunable conductances (sodium, potassium, and chloride). By systematically varying both the intrinsic parameters and the extracellular potassium level, we map the resulting activity patterns and bifurcation sequences. Our approach allows us to identify parameter sets that yield equivalent dynamical transitions—e.g., from tonic spiking to bursting or depolarization block—determine how sensitivity to external perturbations depends on intrinsic configuration, and characterize how degeneracy evolves under changing environmental constraints.

This analysis provides insight into the interplay between internal variability and external modulation in shaping neuronal dynamics. While degenerate configurations may exhibit equivalent behavior under baseline conditions, they may diverge in their responses to perturbations—an effect critical for understanding differential treatment responses and drug resistance in epilepsy [24]. By clarifying how different parameter sets produce similar or divergent transitions, our findings help elucidate mechanisms underlying pathological activity patterns and suggest strategies for controlling neural excitability based on intrinsic neuronal state.

## 2. Results

### 2.1. Similar dynamics across parametrization

Abnormal patterns of brain activity are a hallmark of several neurological and psychiatric conditions, including epilepsy [25], Parkinson’s disease [26], and schizophrenia [27]. A common electrophysiological feature across these disorders is bursting activity, which consists of alternating periods of rapid neuronal firing and quiescence [8,10]. These bursts—defined as clusters of spikes separated by silent phases—can be categorized into distinct patterns based on properties such as spike amplitude, frequency, and burst duration.

In epilepsy, seizures correspond to specific types of bursting patterns and are identified in both experimental recordings and theoretical models [7,17]. These patterns have been observed across preparations, including in vivo and in vitro studies, and across species, suggesting a conserved underlying mechanism. Importantly, such activity can be understood within the framework of nonlinear dynamical systems, where the transitions into and out of bursting regimes are governed by bifurcations—qualitative changes in the system’s stability structure [10].

In low-dimensional models, especially two-dimensional systems, the majority of bursting patterns relevant to epileptiform activity are associated with specific combinations of onset and offset bifurcations [7,9,17]. These bifurcations are driven by variations in system parameters, such as ionic conductances, that control the transitions between silent and active phases. Mapping these transitions offers a principled way to classify bursting dynamics and understand the mechanisms underlying pathological neural activity.

To generate bursting activity, the system must incorporate additional slow processes that modulate the excitability of the neuron over longer timescales. These slow variables influence the parameters of the fast subsystem, effectively controlling the timing and occurrence of bifurcations at the onset and offset of the bursts [7,10].

The model used in this study captures these dynamics at the level of a single neuron immersed in an external potassium bath and is described by a system of four coupled differential equations [10], see Section 4.1. The fast subsystem consists of the membrane potential and a potassium gating variable, which govern rapid spiking behavior. The slow subsystem includes the intracellular potassium concentration and the extracellular potassium exchange with the surrounding bath. These slow variables evolve on a longer timescale and modulate the fast dynamics, thereby enabling the system to transition between silent and active phases via distinct bifurcation mechanisms.

Importantly, varying the extracellular potassium concentration alters the slow dynamics and can drive the fast subsystem through different onset–offset bifurcation combinations. These bifurcations are typically identified through analysis of the full system, requiring access to both fast variables—in this case, the membrane potential and the gating variable. However, in experimental settings, only the membrane potential is usually recorded. This motivates the need for methods that can infer bifurcation types using membrane potential data alone.

The present model reproduces a variety of bursting patterns observed in epilepsy, and these can be qualitatively classified based on established bifurcation signatures described in the literature [7,10,17]. By comparing model outputs to known dynamical patterns (i.e., bifurcation features), it is possible to identify the likely bifurcation structures underlying seizure-like transitions. This approach enables classification of bursting regimes even in the absence of full access to all model variables, making it applicable to both computational and experimental data. In this work, different types of bifurcations, as shown in Table 1, can be identified based on specific features of the membrane potential trace. Saddle SN and SH bifurcations are distinguished from SNIC bifurcations at the onset or offset of bursting by the presence of a sudden shift in baseline potential or by measuring the difference between the pre-burst baseline and the recovery potential following a spike. In contrast, a SupH bifurcation is identified by a gradual increase (or decrease) in spike amplitude during the onset (or offset) of the burst [7,10,17]. By combining these onset and offset features, we are able to assign each time series to a specific bifurcation category (see Section 4.3).

**Table 1:** Summary of bifurcation types and their characteristic signatures in membrane potential dynamics.

Bifurcation	Acronym	Dynamical signature
Saddle-Node	SN	Abrupt transition with baseline shift between silent and spiking states.
Saddle Homoclinic	SH	Burst termination with diverging inter-spike intervals and sharp offset.
SNIC	SNIC	Continuous frequency reduction; spike rate smoothly approaches zero.
Supercritical Hopf	SupH	Smooth amplitude modulation at onset/offset without baseline discontinuity.

For example, spike trains show SNIC bifurcations at both onset and offset. In seizure-like events (SLE), multiple bifurcation combinations can be observed. The most common patterns are associated with two-dimensional bifurcation sequences such as SN–SH or SN–SupH and SupH–SH. Both tonic spiking (TS) and sustained ictal activity (SIA) are characterized by continuous spiking without an offset phase (here referred to as “infinity offset”). These two patterns can be distinguished by their onset bifurcation: SNIC for TS, and either SN or SupH for SIA. Additionally, depolarization block (DB) can be identified by an initial SN–SupH burst, followed by a silent phase in which the membrane potential remains elevated above the baseline.

In this study, we first manually classified the model-generated time series according to their bifurcation structure. Based on this classification, we then developed a deterministic algorithm capable of qualitatively identifying bifurcation types using only the membrane potential (see Section 4.3). This allowed us to systematically explore the range of dynamical patterns generated by the single-neuron model as its biophysical parameters varied. In particular, we aimed to determine whether different parameter combinations could give rise to the same onset–offset bifurcation structure—that is, to explore degeneracy in the model’s dynamical behavior. These manually labeled features (see Section 4.2) served as the foundation for developing an automated classification algorithm, which enabled the systematic analysis of bifurcation patterns across the full set of simulations.

We conducted an initial set of simulations comparing the output of two distinct parameter sets under varying conditions, including changes in extracellular potassium concentration and applied current. We then extended this analysis by systematically varying the three maximal conductances in the model, along with the extracellular potassium level (as summarized in Table 2). This produced a dataset of 10,000 distinct model realizations, allowing us to study how degeneracy emerges and changes across different regions of parameter space.

**Table 2:** Parameter ranges and number of points for $g_{Na}$, $g_{Cl}$, $g_{K}$, and $[K{]}_{bath}$.

Parameter	$g_{Na}$	$g_{Cl}$	$g_{K}$	$[K{]}_{bath}$
Lower value	26.67	5	14.7	7.5
Upper value	53.33	10	29.33	17.5
Number of points	10	10	5	20

### 2.2. Degenerate parameters, shared patterns

Biophysical models of neural activity are defined by multiple parameters that represent key cellular properties. Among these, conductance parameters typically reflect average values derived from the many voltage-gated ion channels present in a single neuron [28]. These ion channels can dynamically modify their electrophysiological characteristics through regulated synthesis and degradation, replacing existing channels with newly produced variants that differ in subunit composition, localization, or post-translational modifications [2]. Such temporal fluctuations, combined with intrinsic variability across neurons, necessitate analyzing models over a broad range of conductance parameter combinations.

Variations in conductance values can significantly alter the complex mechanisms underlying neuronal electrophysiological behavior [3,5,29]. Different parameter sets, representing distinct internal neuronal configurations, may produce similar activity patterns under certain external conditions. However, when external conditions change, these internal differences can lead to qualitatively different neuronal responses (Fig 1), although in some cases, comparable outputs emerge despite parameter variability, illustrating degeneracy.

**Fig 1:**
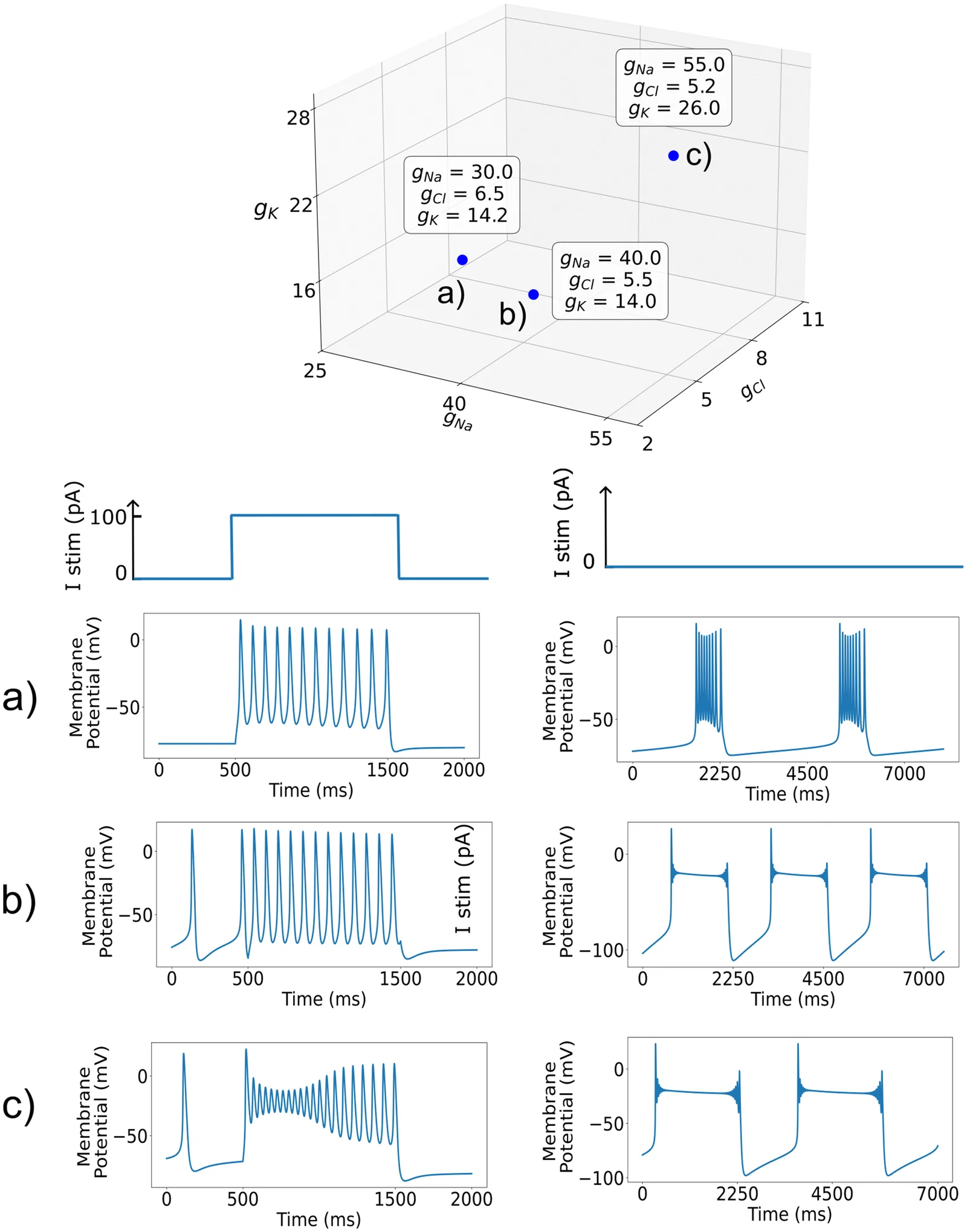
Membrane potential time series simulated under two different potassium bath concentrations and conductance parameter sets. Top panel: Three points are selected in the $\left(g_{\text{Na}},g_{\text{Cl}},g_{\text{K}}\right)$ parameter space. First column: At a potassium bath concentration of 4.5 mM, the neuron is in a resting state and is stimulated with a square-shaped injected current pulse of 100 pA. The first parameter sets produce similar membrane potential responses while the one on the right is different. Second column: At a higher potassium bath concentration of 12.5 mM, no external current is applied. The system exhibits spontaneous bursting activity, with qualitatively different dynamics: the left trace corresponds to a saddle-node/saddle-homoclinic (SN-SH) onset-offset bifurcation pattern, while the middle and the right trace correspond to a saddle-node/ Supercritical | Supercritical Hopf/saddle-homoclinic (SN-SupH|SupH-SH) bursting mechanism.

Our analysis revealed that, even at a fixed extracellular potassium concentration, the model can exhibit a variety of activity patterns. This indicates that degeneracy observed under certain conditions can break down under others. Using the second set of simulations, we investigated how conductance parameter combinations that produce identical onset–offset bifurcations are distributed within the conductance space. For each fixed level of extracellular potassium ([*K*]_bath_), we examined the distribution of pattern types across conductance values.

At the two potassium concentrations shown in Fig 2a and 2b, up to eight distinct activity patterns were observed (Fig 2c).

**Fig 2:**
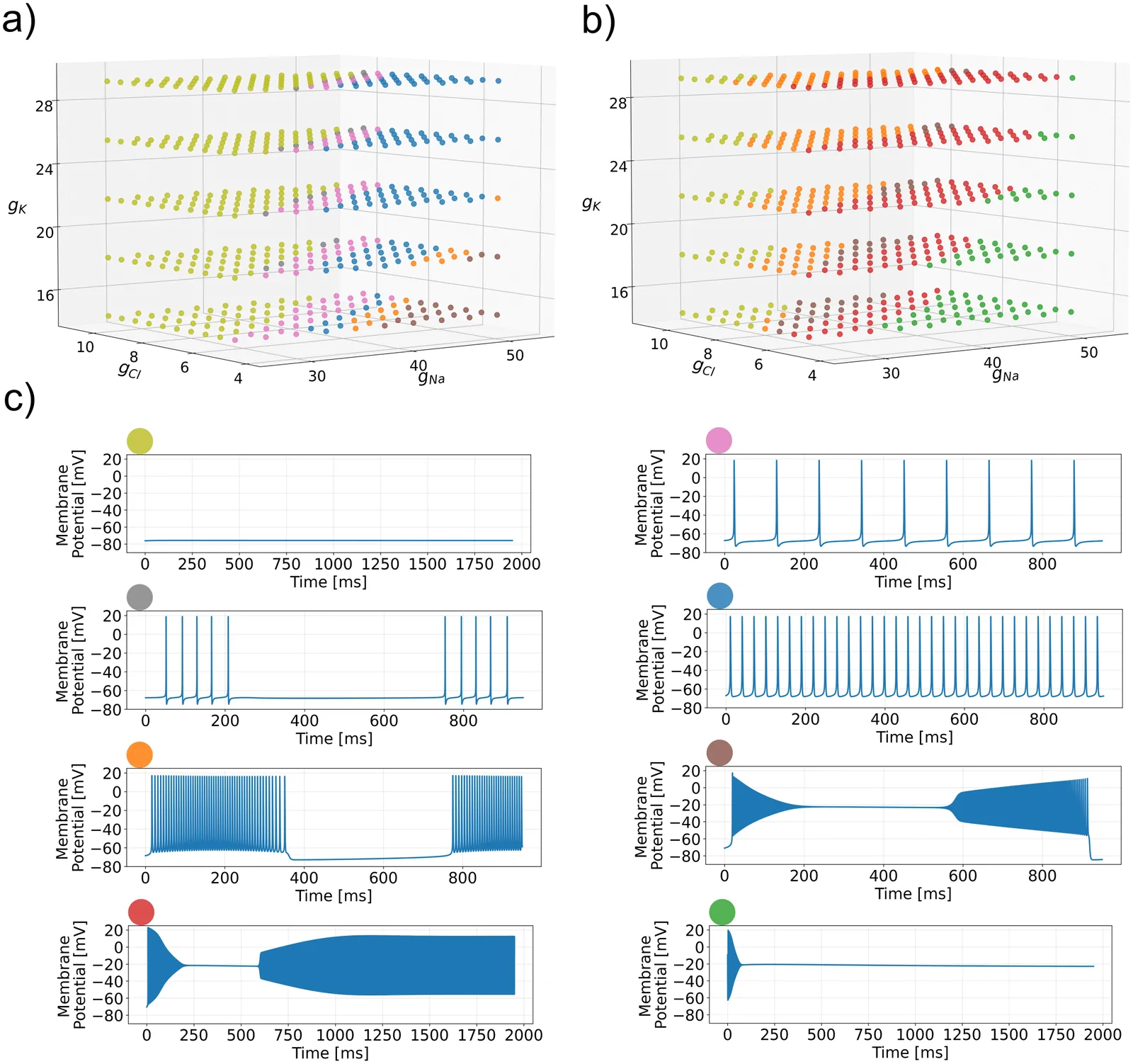
Simulation results at two fixed external potassium bath. Three-dimensional plot in the conductance space at fixed potassium concentrations of 7.5 mM and 17.5 mM. Each point corresponds to a simulation of the model and is colored according to the pattern observed in the corresponding time series. The identified categories are: SNIC–SNIC (grey); SNIC–Infinity (pink); SN–Infinity (blue); SN–SupH|SupH–Infinity (red); SN–SupH (dark green); SN–SH (orange); and SN–SupH|SupH–SH (brown). Light green points correspond to simulations in which no signal emerges from the time series. The corresponding membrane potential traces are shown in panel **(c)**.

Parameter sets that produced the same pattern were found to be closely grouped, suggesting that these neuronal configurations share similar intrinsic properties. Within each pattern category, no further sub-clustering was detected, indicating the presence of compact degeneracy regions in conductance space—defined as connected subsets of parameters that give rise to the same pattern. We then examine how these degeneracy regions change across different levels of extracellular potassium. In this study, we distinguish between pattern classes and degeneracy regions, which describe different but related aspects of the system’s dynamics. A pattern class refers to a specific sequence of bifurcations, such as SNIC–SH or SN–SupH, that defines the qualitative features of neuronal activity under given external conditions. These classes are determined by the model parameters but do not uniquely identify them; in other words, different combinations of intrinsic conductances can give rise to the same pattern class. These combinations form a degeneracy region, which is the subset of parameter space where all points generate the same type of dynamical behavior. While both concepts describe how neuronal activity is organized, their roles differ: pattern classes describe the type of dynamics expressed by the system, whereas degeneracy regions capture the structural variability—at the level of conductance parameters—that supports those dynamics. Distinguishing between these two helps clarify how different neuronal configurations can exhibit similar activity under specific conditions, and how their responses may diverge when external factors change.

Previous studies have shown that increasing extracellular potassium concentration in neurons with fixed conductances can lead to qualitative changes in activity patterns [9]. Here, we investigate whether this effect is uniform across different combinations of conductance parameters. At the highest potassium concentration tested, new activity patterns emerged that were not present at lower levels, while others disappeared (Fig 2b).

Although the degeneracy regions remained compact (see S1 Fig), their distribution across pattern categories differed from that observed at lower potassium levels (Fig 2a). This indicates that the organization of degeneracy regions is sensitive to changes in external conditions.

Overall, our results show that both intrinsic neuronal properties and external ionic concentrations jointly determine the activity patterns exhibited by the model. Increasing potassium modulates the number and identity of conductance configurations associated with each type of pattern. In particular, the proportion of patterns typically associated with pathological activity increases with higher potassium levels, while some patterns observed under baseline conditions are no longer reproducible at elevated concentrations. These shifts suggest that the structural similarity within degeneracy regions is condition-dependent: under varying external conditions, internal variability gives rise to different pattern distributions [3,6]. In the biological system, this corresponds to changes in neuronal conductance profiles, while in the model, it is reflected by altered compositions of the degeneracy regions.

### 2.3. External conditions shape neuronal responses

Transitions between activity pattern categories do not occur uniformly across all neuronal configurations. While similar intrinsic properties may underlie pattern degeneracy under fixed external conditions, differences in these properties can result in divergent responses when the extracellular potassium concentration changes. To fully characterize each neuronal realization, it is necessary to examine its behavior across the entire range of potassium concentrations and assess how the expression of activity patterns varies with these changes.

Across simulations, model realizations exhibit different numbers of distinct activity patterns as the extracellular potassium level increases. Neurons that express the same number of patterns (Fig 3) do not necessarily form compact regions in conductance space, unlike the degeneracy regions observed at fixed potassium levels. The only clearly delineated group consists of parameter sets that fail to generate any activity across the full potassium range—these form a well-defined region in parameter space.

**Fig 3:**
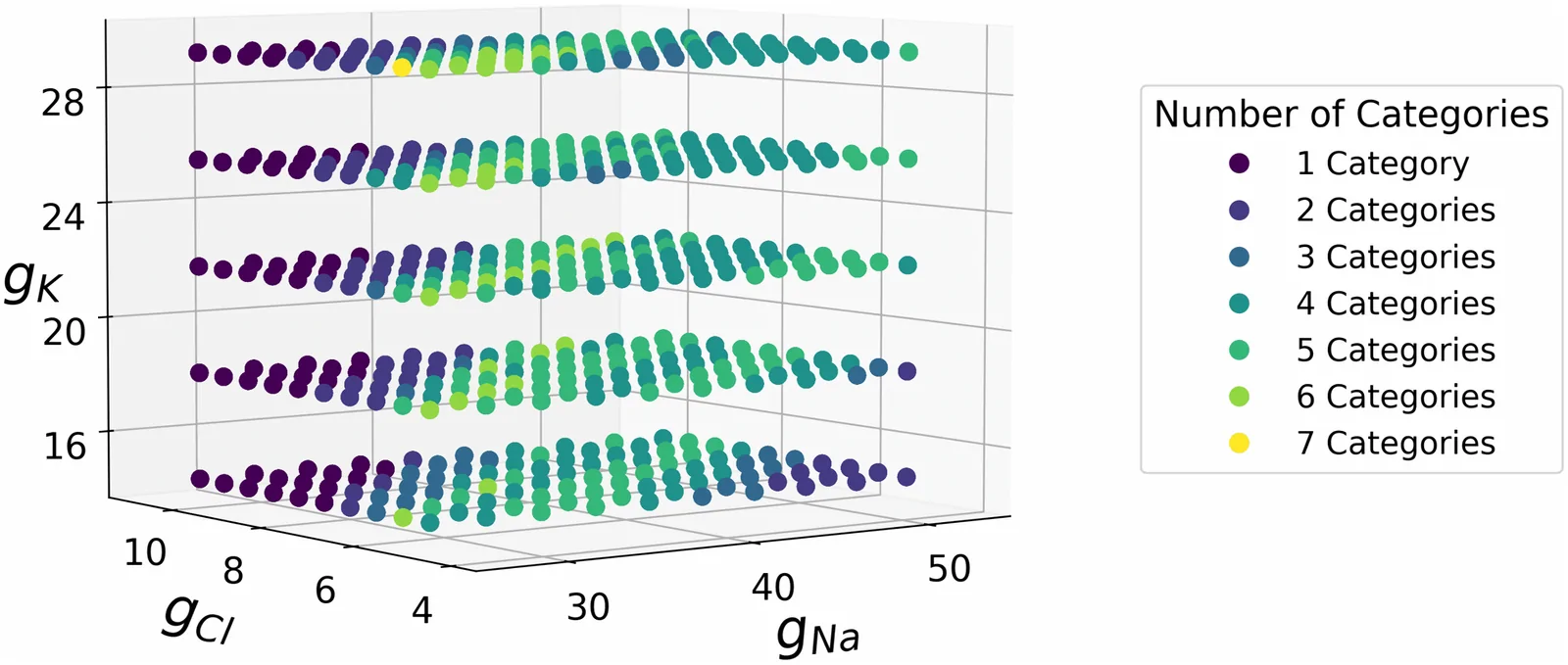
Representation of the number of crossing categories through the potassium bath in parameter space. Three-dimensional plot where each point represents a value in the conductance space, simulated for multiple potassium bath levels within a range from 7.5 to 17.5 mMol. The colors of the points indicate the number of distinct categories that each point can exhibit within the considered potassium range.

This finding implies that neurons exhibiting the same activity pattern under a given condition may diverge when external conditions change. Furthermore, neurons that follow the same sequence of pattern transitions (i.e., identical paths in pattern space) may still differ in the specific potassium concentrations at which these transitions occur. As shown in Fig 4, two parameter sets may display identical patterns up to a certain potassium level, after which they diverge and express different behaviors. For instance, the model realization corresponding to point 1 remains silent across all potassium levels. In contrast, points grouped as 2, 3, 4, 5 and 8, 9 exhibit identical sequences of patterns but over different potassium intervals. Other neurons, such as those labeled 6 and 7, initially express similar patterns at low potassium levels, but then diverge into different pattern categories as potassium increases. The nine points used here can be visualized in Supplementary Materials (see S2 Fig).

**Fig 4:**
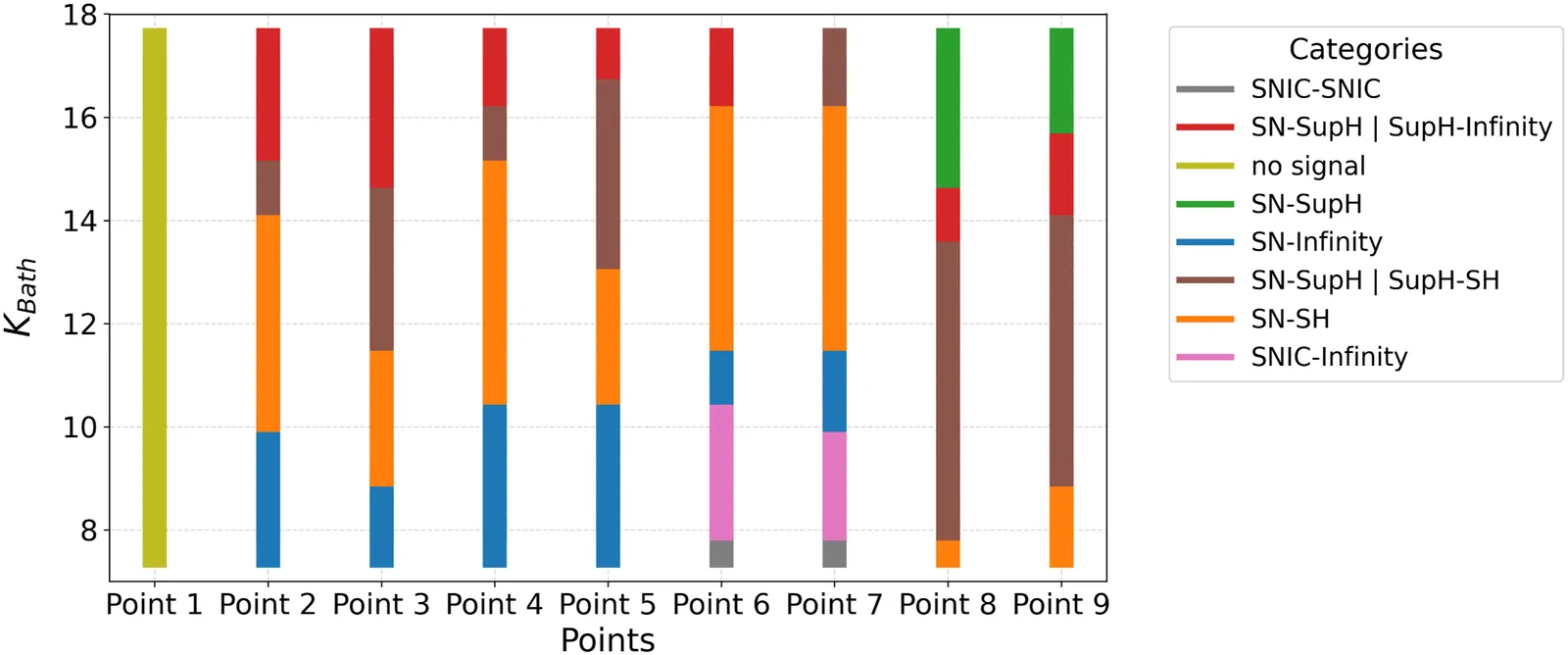
Transition paths among categories across the analyzed potassium range for 9 distinct points in the conductance space. In the colorbars, each color denotes a specific activity pattern, while the length of the colored segment represents the external concentration interval over which the neuron exhibits that pattern.

These results demonstrate that different conductance configurations can lead to distinct dynamical responses, even when they produce the same pattern under baseline conditions. More broadly, the transition between activity categories is not fixed and can vary with external potassium concentration. Even when the pattern trajectory remains the same, the specific potassium levels required to induce transitions between patterns may differ.

In summary, this variability suggests that each point in conductance space has a unique sensitivity profile to external perturbations. Therefore, to better understand the mechanisms underlying such differences, we next investigate which specific conductance features contribute to greater or lesser resilience—defined here as the ability of a given realization in parameter values, to resist changes in activity pattern as extracellular potassium concentration increases.

### 2.4. Resilience reflects ion channel configuration

Because of intrinsic differences, neurons may exhibit the same pattern class at different levels of extracellular potassium concentration ($[K{]}_{bath}$). We define the resilience of a neuron as its resistance to changes in activity pattern when external conditions are modified. Variability in resilience reflects how changes in internal properties—such as ionic conductances—influence the neuron’s ability to maintain its current behavior. The goal of this analysis is to identify which intrinsic conductances most strongly determine this resilience.

In our model, we hypothesize that certain conductances, in particular potassium conductance, have a greater influence on resilience than others. Since the activation and termination of bursts are controlled by potassium fluxes between the external bath and the extracellular space, we expect that the potassium conductance plays a dominant role in determining how much $[K{]}_{bath}$ must increase to induce a pattern transition.

To test this, we conducted the following analysis: for each point in conductance space that expresses a specific pattern class, we recorded the minimum value of $[K{]}_{bath}$ required to elicit that pattern. Then, we calculated the difference in this minimum value between each point and its nearest neighbors along each axis of the three-dimensional conductance space (Fig 5a). For each non-boundary point, this yielded a vector whose components reflect the change in resilience with respect to each conductance direction (Fig 5b). The projections of these vectors onto the corresponding axes reveal which conductances have the greatest influence on resistance to external perturbations (Fig 5c). Although some outliers were observed, these likely arise from classification inaccuracies, particularly near transitions between pattern classes where bifurcation features become difficult to distinguish. Consequently, our interpretation focused on the mean robustness vector, which provides a more reliable representation of the overall trends.

**Fig 5:**
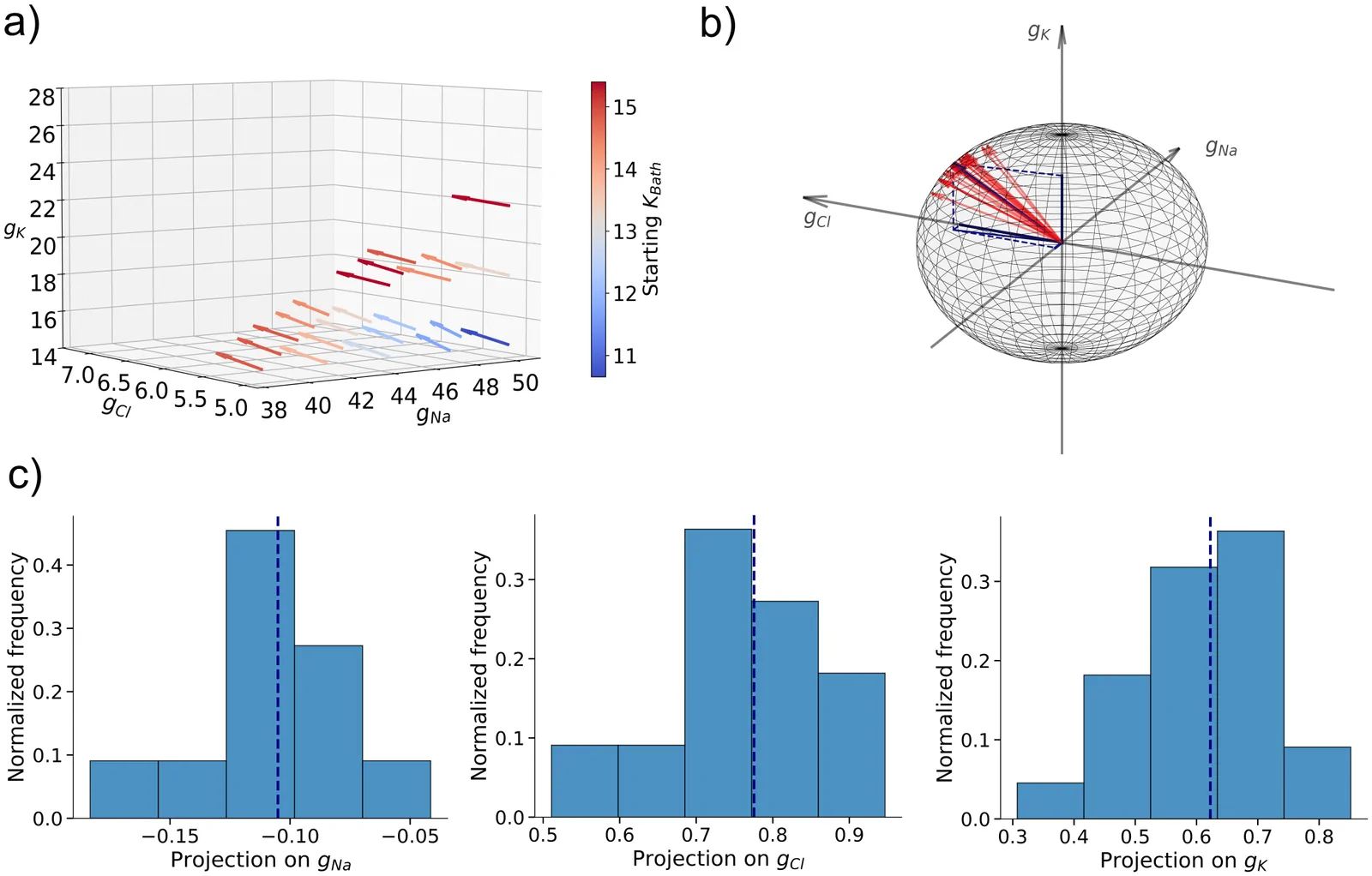
Vectors associated with increasing resilience in conductance space. (a) Vectors corresponding to the largest increase in resilience with respect to the initial conductance configuration. The color scale indicates the minimum bath potassium concentration, ($[K{]}_{bath}$), required to induce a depolarization block (DB). **(b)** Spherical representation of the normalized resilience vectors (red) in the three-dimensional conductance space defined by ($g_{Na}$), ($g_{Cl}$), and ($g_{K}$). The mean resilience vector is shown in blue. **(c)** Distributions of the projections of the normalized resilience vectors onto each conductance axis. The dashed line indicates the corresponding projection of the mean resilience vector.

The analysis of the robustness-vector projections reveals that chloride conductance ($g_{Cl}$) provides the largest contribution to resilience, followed by potassium conductance ($g_{K}$). Both components are positive, indicating that increasing these conductances shifts the bifurcation boundary towards larger variations of extracellular potassium concentration, thereby increasing resistance to transitions between activity patterns. In contrast, sodium conductance ($g_{Na}$) shows a smaller and negative contribution, indicating that increasing sodium conductance slightly reduces robustness under the perturbations considered.

Importantly, this result should not be interpreted as a measure of the absolute contribution of each ionic current or as a direct indication of physiological channel importance. Instead, the robustness vectors quantify how variations in conductance values modify the position of the bifurcation boundary. In conductance-based models, transitions between activity patterns depend on the geometry of the dynamical system, and in particular on how ionic conductances modify the voltage nullcline and the interaction between the fast and slow variables.

The different contributions of ionic conductances are also related to the specific assumptions of the present model. Potassium concentration dynamics are explicitly included, whereas chloride concentration is kept constant. Consequently, the chloride reversal potential ($E_{Cl}$) remains approximately unchanged during variations of extracellular potassium concentration. In contrast, the potassium reversal potential ($E_{K}$) dynamically changes as potassium concentrations evolve. Under the initial conditions of the model, $E_{K}$ is more negative than $E_{Cl}$ ($E_{K}\approx -90$ mV and $E_{Cl}\approx -83$ mV). However, increasing extracellular potassium shifts $E_{K}$ towards less negative values, progressively reducing the potassium driving force near the transition (see S5 Fig). Therefore, although potassium has a major role in the regulation of excitability, variations in $g_{K}$ become less effective in displacing the voltage nullcline close to the bifurcation point. Conversely, because the chloride gradient remains unchanged, variations in $g_{Cl}$ produce a larger displacement of the voltage nullcline in this regime, resulting in the observed ranking.

This interpretation is consistent with a growing body of work showing that ionic concentration dynamics can influence neuronal excitability by modifying the underlying dynamical structure of the system. Allowing ionic concentrations to evolve dynamically introduces slow feedback mechanisms that alter reversal potentials and couple membrane activity with slower homeostatic processes, thereby influencing neuronal dynamics across multiple timescales [30]. Such ion-homeostasis mechanisms can also reshape bifurcation structures and give rise to bistable regimes that are absent when ionic concentrations are treated as fixed parameters [31]. In particular, extracellular potassium accumulation represents a key mechanism through which neuronal activity can be modulated, as activity-dependent changes in potassium levels can alter firing patterns and modify the excitability landscape of neurons [32]. The present results fit naturally within this perspective: the robustness measure does not quantify the strength of individual ionic currents, but rather how variations in conductances influence the location of the transition boundary generated by the interaction between membrane dynamics and ionic homeostasis.

The smaller and negative contribution of $g_{Na}$ should also be interpreted in relation to the model formulation. Because of the electroneutrality assumption, sodium concentration changes are coupled to potassium concentration changes. Therefore, variations in sodium concentration occur in the opposite direction to potassium variations rather than representing an independent perturbation. This coupling affects the sign of the sodium contribution to robustness and explains why increasing $g_{Na}$ produces a small reduction of the robustness measure in the present model.

Overall, these results indicate that neuronal robustness emerges from the interaction between ionic gradients, conductance values, and bifurcation geometry. The observed ranking does not represent a universal hierarchy of ionic channel importance, but rather reflects how different conductances influence the position of the bifurcation boundary within the specific ionic homeostasis framework considered here.

## 3. Discussion

In this study, we developed a method to classify neuronal time series based on features of their bursting dynamics, with the goal of identifying characteristic onset–offset bifurcation types. These features correspond to bifurcations typically found in low-dimensional dynamical systems [7,10,17]. Unlike classical bifurcation detection techniques—which require full knowledge of all system variables [33,34]—our method relies solely on the membrane potential, making it applicable to experimentally recorded data. An important aspect of this work is that both pattern classification and the identification of degeneracy regions are performed using only the membrane potential ($V_{m}$), which is typically the only variable accessible in experimental recordings. Unlike in computational models, where the full set of internal variables and parameters (e.g., ionic conductances, gating variables) is known, experimental data rarely provide direct access to the underlying biophysical configuration. As a result, the pattern class in this study is defined operationally based on features extracted from the membrane potential—such as spike amplitude evolution and baseline shifts—rather than from direct bifurcation analysis involving all system variables. Likewise, degeneracy regions here refer to subsets of parameter space inferred to produce the same voltage-based pattern, even though the full dynamical system structure remains hidden in real biological neurons. This distinction reinforces the relevance of the proposed method for experimental contexts: it provides a framework to classify activity patterns and explore their parametric origins using only observable voltage traces, thereby offering a bridge between theoretical models and electrophysiological data.

We applied this method to a single-compartment neuron model and systematically varied its intrinsic properties by tuning three maximal ionic conductances (sodium, potassium, and chloride), while holding the external potassium concentration fixed. We found that different combinations of conductances could give rise to the same activity pattern and bifurcation sequence. This observation is a manifestation of degeneracy: the capacity of structurally distinct configurations to produce functionally equivalent outputs [1].

Degeneracy has been widely observed in models of the nervous system, particularly in conductance-based models [3,6,35]. Previous studies have also shown that neuronal activity patterns emerge from the interaction between intrinsic membrane properties and ionic concentration dynamics [32,36]. Building on these observations, our results extend the analysis of degeneracy to the level of dynamical transitions by showing that conductance configurations producing equivalent bifurcation sequences can occupy structured regions of parameter space and display distinct responses to extracellular perturbations. Specifically, we show that a given epileptiform activity pattern, such as bursting, can emerge from multiple, topologically compact regions in conductance space. Within each region, certain conductances are tightly constrained to support the observed dynamics, while others exhibit wider variability, consistent with previous reports of parametric compensation in ionic currents [35]. To investigate how these degenerate configurations respond to changes in the external environment, we then varied the extracellular potassium concentration. This manipulation mimics a well-known pathological driver of excitability shifts in epilepsy and other disorders. While degeneracy was preserved under this perturbation, the internal conductance profiles supporting each pattern were redistributed. In particular, the conductance values that led to similar dynamics at baseline often diverged in their reactivity to increasing potassium levels.

To quantify this divergence, we introduced the notion of resilience, defined as the minimum extracellular potassium concentration required to elicit a given activity pattern from a particular conductance configuration. This metric captures the threshold for transition into a specified regime (e.g., from tonic firing to bursting) under external drive. Importantly, it allows us to evaluate not just whether a configuration supports a pattern, but how sensitive it is to perturbations in the extracellular environment. Using a directional analysis across the three conductance axes, we estimated local gradients of resilience and visualized these changes using a spherical vector representation. Our findings show that, within the explored model and perturbation regime, chloride and potassium conductances contribute strongly to shaping resilience, while sodium has smaller and antisymmetric effects.

These results reveal that degeneracy is not a static property: even if multiple conductance configurations are equivalent under fixed conditions, their trajectories under external modulation may diverge. Thus, degeneracy coexists with structured variability in how neurons respond to environmental change. This has important implications for both modeling and interpretation of electrophysiological data.

Our approach, while targeted and informative, involves a number of simplifications. We varied only three maximal conductances and kept all other parameters—including those controlling interactions with the extracellular space—constant. The conductances used represent averaged properties of ion channels rather than detailed channel kinetics, limiting the biological variability captured. Moreover, our model includes only potassium as the extracellular modulator, excluding other ion concentrations, synaptic input, or metabolic effects. These constraints narrow the range of observed mechanisms and the possible manifestations of resilience.

In larger models with more dimensions, the degeneracy landscape becomes increasingly complex and difficult to visualize. In such cases, methods from machine learning, especially Bayesian inference, can support structured exploration of parameter space, quantify uncertainty, and identify high-dimensional degeneracy manifolds [37]. We have proposed a framework to identify and analyze degeneracy in neuronal activity patterns, particularly those relevant to epileptic dynamics. Our method enables the classification of bursting regimes using membrane potential alone and introduces a metric—resilience—to quantify how internal configurations modulate a neuron’s response to extracellular perturbations.

By varying conductances in a simplified neuron model, we confirmed the presence of degeneracy across multiple bifurcation types under fixed external conditions. We further demonstrated that while degenerate configurations support similar patterns at baseline, they differ in how they transition under changes in potassium concentration. This dual analysis of pattern robustness and sensitivity provides a new way to study intrinsic variability in neurons.

These findings have broader implications for experimental neuroscience. Electrophysiological recordings often reveal functionally similar neurons with diverse molecular or biophysical profiles. Our results suggest that comparing neurons solely based on molecular markers is insufficient [38]; instead, their responses to perturbations offer a richer basis for classification [39]. This is also relevant at the network level, where intrinsic conductance variability has been shown to affect dynamics more than synaptic weights.

Ultimately, a deeper understanding of degeneracy and resilience at the single-neuron level could inform personalized models of brain function and dysfunction. In epilepsy, in particular, variability in single-neuron resilience may contribute to heterogeneous responses to treatment and drug resistance [24]. Our approach provides a principled basis for such investigations and a step toward the integrative modeling of neuronal variability across scales.

## 4. Materials and methods

### 4.1. Model

In this study, we adopt a single level neuron model that describes the electrophysiological dynamics of a neuron immersed in an extracellular potassium bath. The model is formulated as a system of four coupled ordinary differential equations forming a slow-fast dynamical system.


$$\begin{array}{c} \frac{dV}{dt}=\left(-\frac{1}{C_{m}}\right)\left(I_{\text{Cl}}+I_{\text{Na}}+I_{\text{K}}+I_{\text{pump}}\right) \end{array}$$ (1a)

$$\begin{array}{c} \frac{dn}{dt}=\frac{n_{\infty }-n}{\tau_{n}} \end{array}$$ (1b)

$$\begin{array}{c} \frac{d\Delta [K{]}_{i}}{dt}=-\frac{\gamma }{w_{i}}\left(I_{\text{K}}-2I_{\text{pump}}\right) \end{array}$$ (1c)

$$\begin{array}{c} \frac{d[K{]}_{g}}{dt}=\varepsilon \left([K{]}_{\text{bath}}-[K{]}_{o}\right) \end{array}$$ (1d)

Eq. 1a and Eq. 1b, describing the membrane potential and the gating variable for potassium conductance, respectively, constitute the fast subsystem. In contrast, the slow subsystem is defined by Eq. 1c and Eq. 1d, which describe the dynamics of intracellular potassium concentration and extracellular potassium buffering by the external bath, respectively. The ionic currents are given by the following expression:


$$\begin{array}{c} I_{\text{Na}}=\left(g_{\text{Na,l}}+g_{\text{Na}}m_{\infty }(V)h(n)\right)\left(V-26.64\log\left(\frac{[\text{Na}{]}_{o}}{[\text{Na}{]}_{i}}\right)\right) \end{array}$$ (2a)

$$\begin{array}{c} I_{\text{K}}=\left(g_{\text{K,l}}+g_{\text{K}}n\right)\left(V-26.64\log\left(\frac{[K{]}_{o}}{[K{]}_{i}}\right)\right) \end{array}$$ (2b)

$$\begin{array}{c} I_{\text{Cl}}=g_{\text{Cl}}\left(V+26.64\log\left(\frac{[\text{Cl}{]}_{o}}{[\text{Cl}{]}_{i}}\right)\right) \end{array}$$ (2c)

$$\begin{array}{c} I_{\text{pump}}=\rho \frac{1}{1+\exp\left(\frac{1}{2}(21-[\text{Na}{]}_{i}\right))}\frac{1}{1+\exp\left(5.5-[K{]}_{o}\right)} \end{array}$$ (2d)

Eq. 2a, Eq. 2b, and Eq. 2c describe the ionic currents flowing through the voltage-gated sodium, potassium, and chloride channels, respectively. Eq. 2d represents the current generated by the sodium-potassium pump. The conductance terms in these equations depend on the membrane potential *V* and the gating variable *n*, and are defined in Eq. 3a, Eq. 3b and Eq. 3c:


$$\begin{array}{c} m_{\infty }(V)=\frac{1}{1+\exp\left(\frac{-24-V}{12}\right)} \end{array}$$ (3a)

$$\begin{array}{c} n_{\infty }(V)=\frac{1}{1+\exp\left(\frac{-19-V}{18}\right)} \end{array}$$ (3b)

$$\begin{array}{c} h(n)=1.1-\frac{1}{1+\exp\left(-8(n-0.4)\right)} \end{array}$$ (3c)

The initial values for the dynamical variables and the ionic concentration are shown in Table 3.

**Table 3:** Initial values of the model variables.

Variable	Initial Value
*V*	-70 mV
*n*	$n_{\infty }(-70)$
$\Delta [K{]}_{i}$	0
$[K{]}_{g}$	0
$[K{]}_{o}$	[*K*]_0,*o*_
$[K{]}_{i}$	[*K*]_0,*i*_
$[Na{]}_{i}$	[*Na*]_0,*i*_
$[Na{]}_{o}$	[*Na*]_0,*o*_
$[Cl{]}_{i}$	[*Cl*]_0,*i*_
$[Cl{]}_{o}$	[*Cl*]_0,*o*_

The initial conditions for the ionic concentrations were chosen to reflect physiologically plausible values commonly used in computational models of neuronal dynamics. Specifically, the initial intracellular sodium concentration was set to $[\text{Na}{]}_{i,0}=16.0$ mM, while the extracellular sodium concentration was $[\text{Na}{]}_{o,0}=138.0$ mM. For potassium, the intracellular and extracellular concentrations were initialized to $[K{]}_{i,0}=140.0$ mM and $[K{]}_{o,0}=4.80$ mM, respectively. Chloride concentrations were also included, with an extracellular value of $[\text{Cl}{]}_{o,0}=112.0$ mM and an intracellular value of $[\text{Cl}{]}_{i,0}=5.0$ mM. At each timestep, the ionic concentrations vary as follow:


$$\begin{array}{c} \Delta [\text{Na}{]}_{i}=-\Delta [K{]}_{i} \end{array}$$ (4a)

$$\begin{array}{c} \Delta [\text{Na}{]}_{o}=-\beta \;\Delta [\text{Na}{]}_{i} \end{array}$$ (4b)

$$\begin{array}{c} \Delta [K{]}_{o}=-\beta \;\Delta [K{]}_{i} \end{array}$$ (4c)

$$\begin{array}{c} {}[K{]}_{i}=[K{]}_{i0}+\Delta [K{]}_{i} \end{array}$$ (4d)

$$\begin{array}{c} {}[\text{Na}{]}_{i}=[\text{Na}{]}_{i0}+\Delta [\text{Na}{]}_{i} \end{array}$$ (4e)

$$\begin{array}{c} {}[\text{Na}{]}_{o}=[\text{Na}{]}_{o0}+\Delta [\text{Na}{]}_{o} \end{array}$$ (4f)

$$\begin{array}{c} {}[K{]}_{o}=[K{]}_{o0}+\Delta [K{]}_{o}+\Delta [K{]}_{g} \end{array}$$ (4g)

The conductance parameters and the potassium concentration were varied in the intervals shown in Table 2, while the other parameters are fixed at values in Table 4.

**Table 4:** Model parameters fixed.

Parameter	Symbol	Value
Conversion factor	γ	$0.04\;\text{mol}/(\text{C}\cdot \mu {\text{m}}^{3})$
Diffusion time constant	ε	0.01 ms^–1^
Membrane capacitance	$C_{m}$	1 nF
Gating time constant	$\tau_{n}$	0.25
Intracellular volume	$w_{i}$	$2160\;\mu {\text{m}}^{3}$
Extracellular volume	$w_{o}$	$720\;\mu {\text{m}}^{3}$
Intra/extra cellular volume ratio	β	3
Maximal Na/K pump current	ρ	250 pA
Sodium leak conductance	*g*_Na,l_	0.02 nS
Potassium leak conductance	*g*_K,l_	0.12 nS

The simulations themselves are based on the integration of the system of differential equations using the Runge-Kutta method. They are performed under fixed initial conditions and span a time range of 10,000 ms. This approach ensures that the dynamic behavior is accurately captured for each set of parameters.

### 4.2. Manual targeting procedure of onset-offset bifurcations

In this work, we first developed a Python tool to classify bifurcations by visually estimating the main features related to onset-offset bursts [7,10,17]. According to the model [9], only one pattern (dynamotype) appears in a given time series. For an accurate estimation, we required time series with at least three spikes. We did not consider the first burst because its onset-offset bifurcations can be influenced by the initial conditions of the system.

The manual targeting tool works as follows. It starts from an initial point in a three-dimensional parameter space defined by the three conductances, while keeping the potassium concentration fixed. The tool displays the membrane potential plots on the screen. Next, the user presses the button corresponding to the observed dynamotype. Once a button is pressed, the current parameter values and the selected target are recorded in a data file. After saving the data, the program automatically runs the simulation for the next set of conductance values.

This procedure was repeated for the entire range of conductance values and was applied for each potassium concentration listed in Table 2.

### 4.3. Features based targeting

After manually classifying the time series according to the observed patterns, we collected the most relevant features to develop a deterministic algorithm for classifying the time series. First, we analyzed the time series based on the spikes they emitted. For spike detection, we used the SciPy find peaks function. Initially, we identified all time series with no spiking activity and labeled them as “no signal” time series. For the remaining time series, we grouped spikes into bursts using an algorithm based on the inter-spike distance. For each time series, we computed—when available—the last two bursts. This approach helps us avoid the transient and less accurate behavior often observed in the first burst. For both bursts, we computed the onset and offset features. The full list of features is available on GitHub; here, we only mention those used for bifurcation detection. Next, we divided the ensemble of time series into two main groups based on the presence or absence of the SupH bifurcation. According to the literature, spike amplitude shows an increasing trend at onset and a decreasing trend at offset. Therefore, we calculated the ratio between the spike amplitudes at onset (or offset) and the maximum spike amplitude in the burst. If the amplitude ratio at onset for the last burst is below 0.8, we classify the time series as exhibiting a SupH bifurcation. In cases where the last burst begins just before the end of the time series, we refer to the amplitude ratio of the penultimate burst. The threshold value of 0.8 was selected because it provides a clear qualitative separation between trajectories that progressively recover from a non-excitable fixed point, as expected after a SupH onset bifurcation, and trajectories in which spikes are continuously generated, as in the SN-SH case. Close to the codimension-2 transition between these regimes, the spike amplitude varies continuously and no sharp boundary exists. Consequently, some trajectories near this transition may not be perfectly separable using membrane potential alone. Moreover, in the SN-SH case very small spikes may not be detected by the peak-finding algorithm, slightly reducing the robustness of the amplitude ratio criterion. Since the purpose of this work is a qualitative classification of bursting mechanisms rather than an optimal statistical classifier, we considered this threshold sufficiently reliable. Once we defined the SupH and non-SupH groups, we further distinguished the different pattern categories within each group. Here, the vertical bar notation in labels such as SN-SupH | SupH-SH and SN-SupH | SupH-Infinity denotes a change in the bifurcation mechanism governing successive transitions between the active and silent phases of the bursting dynamics. In particular, in two-dimensional nonlinear systems, a Supercritical Hopf (SupH) bifurcation can occur both at burst onset and burst offset. When a SupH bifurcation occurs at the offset, the system transitions from the spiking regime to a stable non-excitable fixed point. A second SupH bifurcation is then required to destabilize the fixed point and restore oscillatory activity. After this transition, the dynamics can either terminate through a Saddle Homoclinic (SH) bifurcation, leading back to a stable excitable fixed point, or continue in a persistent oscillatory regime associated with an Infinity bifurcation. Therefore, the vertical bar separates the bifurcation mechanisms associated with these successive transitions within the bursting cycle. In the SupH group, if the number of spikes in the last burst exceeds 5000, the classification is SN-SupH | SupH-Infinity. This class corresponds to bursting dynamics in which the oscillatory regime persists without a clear termination, consistently with an Infinity offset bifurcation. The threshold of 5000 spikes was introduced to distinguish these trajectories from depolarization block (DB) cases, where only a single burst is present but its duration remains finite. By requiring a sufficiently large number of spikes, the criterion reliably identifies the effectively unbounded oscillatory activity expected in the Infinity case while preserving a simple deterministic decision rule. For the remaining time series, if the burst is identified as type 1 (i.e., bursts characterized by a progressive reduction in spike amplitude leading to a depolarized quiescent state) or type 2 (i.e., bursts in which spike amplitudes remain approximately constant before burst termination) combined with a variance in the peak amplitude of the last burst less than 20, we classify it as SN-SupH (Depolarization Block). The distinction between type 1 and type 2 bursts was introduced to separate trajectories exhibiting clear depolarization block behavior from those maintaining sustained spike amplitudes near burst termination. All other time series in this group are classified as SN-SupH | SupH-SH. Although these classes become difficult to distinguish close to codimension-2 transitions, this ambiguity reflects the continuous evolution of the underlying dynamics rather than exclusively a limitation of the classifier. We therefore preferred a deterministic rule-based approach, whose decisions can be directly interpreted in terms of bifurcation properties, instead of introducing a supervised classifier with reduced physical interpretability. For the non-SupH group, we used the following procedure. To distinguish time series with not ending spiking activity (Infinity offset) from those without, we computed the ratio of the number of spikes in the penultimate burst to the number in the last burst. If this ratio is less than 1, the time series is classified as Infinity offset. To further differentiate the onset bifurcation in both the Infinity and non-Infinity cases, we combined two features. First, we estimated the baseline jump, defined as the difference between the membrane potential measured just before the start of the burst and its minimum value within the interval defined by the first two spikes. Then, defining the spike period as the time difference between consecutive spikes, we computed the ratio between the first two spike periods to complement the baseline jump. If the baseline jump is less than 0 and the period ratio is greater than 1, the classification is SNIC-SNIC; otherwise, we label it as SN-SH. The use of these features is consistent with the bifurcation characteristics reported in the literature. In fact, a baseline jump occurs at onset (or offset) only for the SN (or SH) bifurcation, and for the SNIC bifurcation, the frequency approaches zero at the bifurcation point, meaning that its inverse (the spike period) must be greater than 1. Accordingly, the baseline jump criterion was introduced because its presence is expected for SN and SH bifurcations but not for SNIC bifurcations. However, close to the saddle-node loop codimension-2 transition connecting these regimes, the baseline jump changes continuously and no sharp separation can be expected. Therefore, the adopted threshold should be interpreted as a qualitative criterion rather than an exact boundary. Finally, we briefly assessed the expected robustness of the proposed rules to additive noise. Small-amplitude noise may slightly modify the firing frequency within a burst or anticipate spike initiation when the system operates close to a bifurcation, but these effects generally do not alter the extracted bursting structure. The main source of uncertainty concerns criteria based on spike amplitudes, since noise may distort very small spikes or prevent their reliable detection, thereby affecting the measured amplitude ratio. A complete quantitative sensitivity analysis would require a dedicated study. For sufficiently large noise amplitudes, the stochastic perturbation alters the underlying dynamical system itself, making a deterministic bifurcation description no longer fully appropriate and requiring a stochastic dynamical systems framework. Finally, the performance of the proposed feature-based deterministic classification algorithm was compared with the manual classification used as reference throughout this work. Overall, the two approaches produced very similar results (see S3 and S4 Figs). Although some individual time series were assigned to different categories, these discrepancies were limited in number and did not alter the qualitative interpretation of the bifurcation structure or the conclusions drawn from the analysis. A detailed comparison between the manual and feature-based classifications, including the cases in which the two methods disagree, is provided in the Supplementary Materials.

### 4.4. Computation and visualization of resilience variations

To assess the resilience of specific regions of the parameter space with respect to the emergence of a given dynamical pattern as the extracellular potassium concentration increases, we adopted the following procedure. First, for each point in the parameter space, we determined the minimum potassium bath concentration required to elicit the selected pattern. This value represents the critical potassium concentration at which the pattern first appears. Next, for each point (excluding those located at the boundaries of the selected region), we computed the differences between its critical potassium concentration and those of its nearest neighbors along each axis of the three-dimensional conductance space. These differences define a vector whose components quantify the local variation in the critical potassium concentration along each conductance axis.

The direction and magnitude of this vector provide information about the local robustness of the pattern. In particular, the vector direction identifies the conductance parameters that have the greatest influence on the onset of the pattern, whereas its magnitude quantifies the overall sensitivity of the critical potassium concentration to local parameter variations. Larger magnitudes indicate greater sensitivity and, consequently, lower robustness to changes in the conductance parameters. To characterize the dominant orientation of these vectors within the conductance space, we normalized each vector and computed its projections onto the three coordinate axes. The normalized projections quantify the relative contribution of each conductance parameter to the robustness vector, thereby revealing, in percentage terms, which conductance exerts the strongest influence on the robustness of the pattern.

## Supporting information

S1 FigTwo-dimensional projections of the SN-SH and SN-SupH degeneracy groups onto the $g_{Na}$-$g_{Cl}$ plane.Two-dimensional projections of the SN-SH and SN-SupH degeneracy groups shown in Fig 2b at fixed potassium concentrations of 17.5 mM. The color indicates the number of points along the hidden $g_{K}$ axis projected onto each point in the $g_{Na}$-$g_{Cl}$ plane.(PNG)

S2 Fig Conductance space points selected for the analysis of transitions between categories. Three dimensional representation of the conductance space, in which each point corresponds to a parameter set simulated across potassium bath concentrations ranging from 7.5 to 17.5 mM. The colored points indicate the number of distinct categories exhibited by each set of parameters over the analyzed potassium range. The nine highlighted points correspond to those examined in Fig 4, where their transition paths between activity categories are shown.
(PNG)

S3 Fig Comparison of three-dimensional conductance space plots obtained using manual and features based targeting at a fixed potassium bath concentration. Three-dimensional representations of the degeneracy in conductance space at a potassium bath concentration of 7.5 mM shown in Fig 2a, comparing the results obtained using manual targeting and features based targeting.
(PNG)

S4 Fig Comparison of resilience vector spherical representations obtained using manual and feature-based targeting. Spherical representations of the normalized resilience vectors (red) in the three-dimensional conductance space, comparing the manual targeting result shown in Fig 5b with the features based targeting result. The corresponding mean resilience vector is shown in blue.
(PNG)

S5 Fig Comparison of chloride and potassium Nernst potentials at two different external potassium bath concentrations. Top p**anel:** Time series representing the imposed potassium bath concentration. During the first half of the simulation, the potassium concentration is set to 5.5 mM; during the second half, it rapidly changes to 8.5 mM. **Bottom panel:** Chloride and potassium Nernst potentials (red dotted and blue solid lines, respectively) as functions of the imposed potassium bath concentration. The chloride potential remains constant, whereas the potassium potential depends on the potassium bath concentration. When the potassium concentration is set to 8.5 mM, the potassium potential becomes less electronegative than the chloride potential.
(PNG)
