# Supplementary material for: Degeneracy in neuronal bifurcation landscapes: Equivalent bifurcation sequences across distinct parameter sets
Source: PLoS Comput Biol. 2026 Oct 30;22(10):e1014893. doi: 10.1371/journal.pcbi.1014893 (PMC13739234; doi:10.1371/journal.pcbi.1014893)
Supplement: Supplementary file 1 [file pcbi.1014893.s007.pdf]

Reviewer #1: The authors investigate the firing patterns of a single-compartment neuron model with three conductances (Na, Cl, Ca) under systematic variation of conductances and extracellular potassium concentration. Elevated extracellular potassium concentration, as observed during epileptic seizures, can induce bursting activity characterized by specific onset and offset bifurcations. The authors classify bifurcation sequences solely from membrane voltage time series. This voltage-only approach is novel, as classical methods rely on additional dynamical variables, and important since experiments typically provide only membrane voltage recordings. Their analysis further identifies regions of degeneracy in which distinct parameterizations produce identical dynamical behavior. They introduce resilience as the minimal extracellular potassium concentration required to induce a given activity pattern and potassium conductance having the largest effect on it.

The manuscript accurately and fairly summarizes prior work, identifies a clear gap, and formulates a well-defined research question that it addresses with a significant contribution. The methods are appropriate and described in sufficient detail to allow reproduction. Given the focused research question, the statistical analysis is adequate and conceptually sound, and the work appears technically correct. Broader generalization would require additional statistics across more initializations and parameterizations. Assumptions are justified and the results support the conclusions without overinterpretation. The discussion places the findings in context and lists limitations. The abstract reflects the content faithfully.

The presentation and language are clear. Figures and tables are generally accurate, clear, and necessary. A particular strength of the study is its logical design and the development of a practical classification tool that may benefit experimental research.

The manuscript is suitable for PLOS Computational Biology, as it advances understanding of epileptic activity through a controlled biophysical model and a novel computational classification approach. The minor revisions suggested below would further improve the manuscript.

Content
* l. 205 Table 1: Why does the number of sampled points differ between parameters? How were the lower and upper bounds of each parameter range determined?
* l. 296 Please mark the nine points in Fig. 4 that are analyzed in detail in Fig. 5.
* l. 600-601 What does the vertical bar in SN-SupH | SupH-SH and SN-SupH | SupH-Infinity denote? Please explain this when introducing the bursting categories.
* l. 598-599 What do "type 1" and "type 2" refer to? Please define these categories at first mention.

Clarity
* Consider summarizing all bifurcation types (abbreviation, full name, key features) in a single table and referring to them thereafter by abbreviation only.
* Please reference figures in order of appearance and place each figure after its first mention (e.g., consider switching the left and right panels of Figs. 2 and 3).
* Consider numbering all displayed equations, as this may facilitate referencing in future work / discussions.
* l. 137-146 Consider referring to Eq. 1a-d already here.
* l. 164-174 An explicit reference to the algorithm in Section 5.3 would be helpful.
* l. 187 An explicit reference to Section 5.2 would be helpful.
* l. 189 Refer to Section 5.3 instead of Section 5.
* l. 195 Refer to Section 5.2 instead of "Materials and Methods."

* l. 224 (Fig. 1) The color bar for K_bath is unnecessary given the two fixed values. Consider labeling the three configurations as (a), (b), (c) and the 3D plot as (d), marking the points directly rather than using arrows. Arranging subplots in columns would avoid repetition of current traces.
* l. 256 In Fig. 2, mark the three points in conductance space corresponding to the membrane traces.
* l. 256, 261 Consider adding a legend to the 3D plots explaining the color coding.
* l. 256, 261 Consider merging Figs. 2 and 3 or separating their discussion more systematically in the text.
* l. 256, 261 Use consistent colors for membrane traces and their corresponding points in conductance space.
* l. 352 In Fig. 6, use subfigure labels (a), (b), (c), (d) instead of a.1, a.2, b1, b2.
* l. 352 Define the abbreviation "DB" earlier or omit since it is used only once.
* l. 547 Table 3 may be placed earlier, as it defines variables in Eq. 1.
* l. 547 Please specify the unit (ms) for the time constant.
* l. 573 Consider a more specific title than "Deterministic classifier algorithm," indicating that it classifies bifurcation types.
* l. 573 Section 5.3 would benefit from a structured algorithm listing with numbered steps and formulas.

Formatting
* l. 205 Table 1 (and elsewhere, including figure labels) Typeset Na, Cl, K, and bath upright in math expressions.
* l. 223 Ensure consistent spacing in figure references (e.g., "Fig. 1").
* l. 224 (caption) "pA" appears to have formatting issues; please correct.
* l. 224 Fig. 1 Label the y-axis as V_m for consistency.
* l. 508 Typeset the differential operator d upright.

Typos
* l. 115 corresponds -> correspond
* l. 224 (Fig. 1 caption) fist -> first
* l. 427 model -> models
* l. 545 conducatance -> conductance

RESPONSE:

Dear Reviewer #1,

Thank you very much for your careful review and for your positive evaluation of our work. We particularly appreciate your interest in improving the clarity and readability of the manuscript. We carefully considered all of your suggestions and revised the manuscript accordingly.

In particular, following your recommendations, we modified the structure of Figure 1 to improve its readability and merged the previous Figures 2 and 3 into a single figure. The revised figure now uses consistent color coding to directly associate the membrane

voltage traces with the corresponding locations in the conductance space, making the relationship between the two representations much clearer.

Regarding your comments on the clarity of the manuscript:

Sampling points and parameter ranges (Table 1).

We chose a smaller number of sampling points for g_K to reduce the total number of simulations, particularly during the initial phase of the study, where all simulations required manual inspection and validation. This reduction was applied only to g_K because, by construction of the model, we expected an inverse relationship between the maximal potassium conductance and the extracellular potassium concentration. Consequently, our primary objective was to investigate the compensatory mechanisms involving the other two conductances, g_Na and g_Cl, while maintaining a computationally manageable dataset.

The parameter ranges were selected as ±50% around the conductance values reported by Depannemaecker et al. (2022), where the model was originally introduced and analyzed using fixed conductance values under different extracellular potassium concentrations. Since the conductances in our simplified model represent generalized ionic conductances rather than the variability of specific channel subtypes, the ±50% interval was chosen as a biologically plausible range capable of generating a broad repertoire of neuronal activity while remaining close to the calibrated parameter set.

As shown by our results, even within this range a subset of parameter combinations did not exhibit spontaneous activity. Expanding the explored ranges further, or shifting them away from the calibrated values, would likely increase the proportion of non-spiking regimes or, conversely, produce an excessive number of depolarization block states, thereby reducing the diversity of dynamical behaviors observed. We have clarified these points in the revised manuscript.

We also addressed the remaining points concerning the presentation and terminology throughout the manuscript. In particular:

* The nine parameter points discussed in detail are now explicitly highlighted as Supplementary Materials (S2 Fig.)

* We introduced a summary table collecting all bifurcation types, their abbreviations, full names, and defining characteristics.

* Figures have been reordered where appropriate, references to figures, equations, and sections have been made consistent throughout the manuscript, and all suggested formatting corrections and typographical errors have been addressed.

Regarding the notation SN–SupH | SupH–SH and SN–SupH | SupH–Infinity, we have added the following clarification when introducing the bursting categories. The vertical bar denotes the transition between two successive bifurcation mechanisms occurring within the same bursting cycle. In two-dimensional nonlinear systems, a Supercritical Hopf (SupH) bifurcation may occur either at burst onset or at burst offset. When the burst terminates through a SupH bifurcation, the system enters a stable non-excitable fixed point. To initiate the next burst, a second SupH bifurcation destabilizes this fixed point and restores oscillatory activity. The subsequent evolution distinguishes the bursting classes: in the SupH–SH case, oscillations terminate through a Saddle Homoclinic (SH) bifurcation, whereas in the SupH–Infinity case they evolve toward a bifurcation at infinity. The vertical bar therefore separates these two successive transitions within the bursting cycle. This explanation has been added to the revised manuscript.

Finally, we clarified the meaning of type 1 and type 2 bursts . Type 1 bursts are characterized by a progressive reduction in spike amplitude leading to a depolarized quiescent state, whereas type 2 bursts maintain approximately constant spike amplitudes until burst termination. Combined with a variance of the peak amplitude during the last burst below the adopted threshold, these criteria allow the classifier to identify SN–SupH (Depolarization Block) dynamics. This definition has now been introduced at its first occurrence in the manuscript and highlighted in the revised algorithm description.

Reviewer #2: General assessment

The authors address a topic of general interest: Are regions of similar spiking behaviour compact sets in parameter space and how are these region influenced by other parameters. This leads to a concrete concept of resilience for spiking neurons. Their study relies on the analysis of a conductance-based model with potassium concentration dynamics. Some of the concrete findings of the article are relatively intuitive and not entirely new and it might be necessary to add some content before the manuscript is suitable for publication.

Comments

The reviewer appreciates the concrete definition of resilience in section 3.4. as the minimum extracellular potassium concentration that elicits a pattern. The gradient of this resilience increase is plotted as vectors for all conductance combinations. This, since not trivial to read, e.g. in Fig. 6, might benefit from additional explanations and visualisation. This is particularly severe, since some of the conclusions are not easily seen in the plots. Contrary to the text (ll. 346), it seems that for high starting K_(Bath) the g_(K) conductance suddenly has smaller contribution. It is difficult to read this plot if

the conductance-axis are scaled differently. This alludes to the more general discussion of how to compare changes in conductances as different as leak, potassium or sodium. The change to polar coordinates does not improve the readability either. This should be improved on.

It might be prudent not to over generalise from simulations with one (to some extend idiosyncratic) model. The statement in ll. 364 “The g_(K) component is consistently positive and larger than the others, indicating that increasing potassium conductance strengthens the neuron’s resistance to transitions in activity patterns.” The authors show only resistance to potassium concentration perturbations, hence it is almost trivially so that the potassium conductance has the strongest influence. The authors discuss this point in ll. 381 onwards. They make a statement about sodium (due to the inverse relation between sodium and potassium, probably referring to equations 4a-g), but show no data. But it would important for the article, to also analyse perturbations in intracellular sodium concentrations or chloride. Is it then still g_(K) or actually g_(Na) that influences resilience. In particular, relations 4a-g rely on the electro-neutrality assumption. Bulk electro-neutrality is locally disobeyed during AP dynamics and in general only eliminates on of the concentration degrees of freedom, see (Hübel and Dahlem 2014) for a more extensive discussion. Considering the importance of the issues for the submitted manuscript it seems necessary to expand on these matters and include a more extensive analysis of sodium currents.

In other words, the consequences of the particular choice of model are unclear and not presented. In particular, the model simplification. For example the instantaneous sodium dynamics, the slaving of the sodium inactivation to the potassium gate are all not really necessary and not motivated.

I have some mild criticism on how the present work in embedded into the existing literature. Some of the statements presented as findings here, are known in older articles. For example in ll. 268 the authors state “our results show that both intrinsic neuronal properties and external ionic concentration jointly determine the activity patterns exhibited by the model”. This, to some extend trivial insight in a non-linear system, has been reported on extensively both in the modelling literature Behbood et al. (2024) as well as in experimental work (Contreras et al. 2021) beyond the work of Eve Marder. The authors could strengthen their manuscript by referring and discussing these older studies.

The semi-manual classification algorithm of onset bifurcation described in the method section, might be exemplified in the results section, showing the reader how it works and providing some statistics on its performance.

Detailed remarks

Please name and introduce all state variables and parameters in the Materials and Methods section 5.1, e.g. what is K_(g).

References

Behbood, Mahraz, Louisiane Lemaire, Jan-Hendrik Schleimer, and Susanne Schreiber. 2024. “The Na+/k+-ATPase Generically Enables Deterministic Bursting in Class i Neurons by Shearing the Spike-Onset Bifurcation Structure.” PLOS Computational Biology 20 (August): e1011751. https://doi.org/10.1371/journal.pcbi.1011751.

Contreras, Susana Andrea, Jan-Hendrik Schleimer, Allan T. Gulledge, and Susanne Schreiber. 2021. “Activity-Mediated Accumulation of Potassium Induces a Switch in Firing Pattern and Neuronal Excitability Type.” PLOS Computational Biology 17 (5): e1008510. https://doi.org/10.1371/journal.pcbi.1008510.

Hübel, Niklas, and Markus A. Dahlem. 2014. “Dynamics from Seconds to Hours in Hodgkin-Huxley Model with Time-Dependent Ion Concentrations and Buffer Reservoirs.” PLOS Computational Biology 10 (12): e1003941. https://doi.org/10.1371/journal.pcbi.1003941.

Hübel, Niklas, Eckehard Schöll, and Markus A. Dahlem. 2014. “Bistable Dynamics Underlying Excitability of Ion Homeostasis in Neuron Models.” PLOS Computational Biology 10 (5): e1003551. https://doi.org/10.1371/journal.pcbi.1003551.

RESPONSE:

Dear Reviewer #2,

Thank you very much for your careful and thoughtful review of our manuscript. We greatly appreciate your detailed analysis of both the scientific content and the interpretation of our results. Your comments prompted us to carefully re-examine our robustness analysis, which ultimately led to substantial improvements in both the presentation and the interpretation of this part of the work.

After recalculating the robustness vectors while properly accounting for the different scales of the three conductances, we obtained results that differ from those reported in the original manuscript. In particular, we found that the dominant contribution to robustness arises from variations in the chloride conductance rather than the potassium conductance. To make this result easier to interpret, we added a new figure showing the projections of the robustness vectors onto each conductance axis. At the same time, we decided to remove the polar-coordinate representation, as we agreed with your observation that it unnecessarily complicated the interpretation and could even be misleading.

Furthermore, we chose to present the robustness analysis only for the Depolarization Block (DB) transitions. Among all activity classes, this category was the least affected by classification uncertainty and therefore provides the most reliable estimate of the robustness vectors. Restricting the analysis to this case substantially improves the readability of both the three-dimensional representation and the corresponding vector projections.

The revised analysis also led us to a different and, in our opinion, more physically grounded interpretation of neuronal robustness. We now relate the observed differences in robustness to the distinct Nernst potentials associated with the different ionic species. Conductances whose reversal potentials are closer to the resting membrane potential exert a stronger influence on the position of the fast-subsystem nullclines. Consequently, the same relative variation in conductance produces a larger displacement of these nullclines, thereby modifying more effectively the distance between the fixed points of the fast subsystem. Since this geometric distance determines how easily an external potassium perturbation can induce a bifurcation, conductances associated with reversal potentials closer to the resting state have a larger impact on the robustness of the neuronal dynamics.

To further support this interpretation, we have included an additional figure in the Supplementary Material (S5 Fig.) illustrating how the relative position of the Nernst potentials explains the greater sensitivity of the nullcline geometry to variations in the chloride conductance. This additional analysis provides a direct mechanistic explanation for the revised robustness ranking.

Regarding your comments on the dependence of our conclusions on the specific model assumptions, we agree that the robustness analysis is necessarily model-dependent. Our goal was not to derive a universal ranking of ionic conductances but rather to introduce a quantitative framework for defining and measuring neuronal robustness within conductance-based models with ion concentration dynamics. We have revised both the Discussion and the corresponding parts of the manuscript to better emphasize this point and to explicitly state the limitations associated with the simplifying assumptions of the model.

Concerning the embedding of our work within the existing literature, we fully agree that several qualitative observations regarding the interplay between intrinsic neuronal properties and extracellular ion concentrations have been reported previously. Accordingly, we revised both the Abstract and the Discussion to better position our contribution with respect to earlier theoretical and experimental studies, including the references you suggested. We now make it clearer that the novelty of the present work lies not in the qualitative observation that intrinsic and extracellular mechanisms jointly shape neuronal activity, but rather in the systematic mapping of conductance space, the voltage-only bifurcation classification framework, and the introduction of a quantitative notion of neuronal robustness based on extracellular potassium perturbations.

Finally, following your suggestion, we expanded the description of the semi-manual classification algorithm (now Features based targeting). Since the classifier is primarily intended as a methodological tool and its performance analysis does not introduce additional biological insights, we chose to present the validation and representative examples in the Methods section and in the Supplementary Material (S3 Fig. and S4 Fig.) rather than in the Results section. We believe that this organization improves the readability of the manuscript while still providing the reader with a clear description of the algorithm and its performance.

Reviewer #3: The manuscript proposes an interesting computational study of "degeneracy" at the level of dynamical transitions in single-neuron models. Using a four-variable slow/fast model with three tunable maximal conductances (gNa, gK, gCl) and an external potassium drive, the authors show that (i) several distinct conductance triplets can elicit the same onset/offset bifurcation sequence and that (ii) these "degeneracy regions'' rearrange when the potassium is varied and also that (iii) the minimal potassium needed to trigger a given pattern ("resilience") depends most strongly on gK. They also describe a pragmatic voltage-only classifier of bifurcation types that could, in principle, be used on experimental traces.

The study is timely, touches on a recurring question in computational neuroscience, and is generally well written. The code is also provided (GitHub is mentioned, and the repository is populated). Nevertheless, it is my view that several conceptual, methodological, and presentation issues should be addressed before the work can be recommended for publication.

Comments:

1) Section 5.3 The deterministic rules (baseline jump, amplitude ratio <0.8, 5000-spike threshold, etc.) are introduced ad-hoc. Please supply a quantitative validation: confusion matrix, precision/recall. A small discussion of the robustness to additive noise would be interesting.

2) How sensitive are the thresholds (0.8)? A short sensitivity analysis, or at least a justification based on theoretical expectations, is needed.

3) It is my understanding that some classes are collapsed (e.g., SN-SupH|SupH-SH) when they cannot be separated by the rules. Is there evidence that they are truly inseparable from Vm only? If not, could a simple supervised classifier (e.g. decision tree) do better?

4) The manuscript alternates between "pattern class'' and "degeneracy region'' but the relationship remains confusing. A degeneracy region is first defined at a fixed potassium bath, yet later the term seems to migrate with the potassium bath. Consider providing a formal definition in the Methods.

5) A 2-D projection (e.g., gNa vs gK with gCl coded by color) showing one complete degeneracy region (or what people call a 'level set'), to convince the reader that it is indeed connected and compact. It could go with one of the 3D plots the authors propose.

6) A short discussion on whether the borders of such regions correspond to codimension-2 bifurcations or to a failure of the classifier would also be interesting.

7) Why is resilience defined only as the minimal potassium bath at which the pattern first appears? Would it not be more informative to track the entire interval of the potassium bath over which the pattern persists?

8) Only three maximal conductances are varied; all time constants, reversal potentials, and pump parameters are frozen. Some discussion of how the conclusions might (or might not) extend to a more biophysically detailed model with, for example, persistent Na+, Ca2+, leak reversal, or dynamic chloride would be welcome.

Minor comments

Citation style oscillates between numeric and author-year. Plos CB uses only numerical

Fig. 1 legend: “fist parameter sets” - first.

The threshold “5000 spikes” looks suspiciously high. Are you sure this is correct?

Spherical coordinates: please check limits, it might be that theta is [0,pi] and not [0, 2pi]

RESPONSE:

Dear Reviewer #3,

Thank you very much for your careful evaluation of our manuscript and for your constructive comments. We particularly appreciate your recognition of the novelty of our computational framework and of the potential applicability of the voltage-only bifurcation classifier. Your suggestions helped us substantially improve both the methodological description and the presentation of our results.

Regarding the deterministic classifier (now Features based targeting), we expanded Section 5.3 to better explain the rationale behind each classification criterion. In particular, we clarified that the thresholds adopted (e.g., the amplitude ratio and baseline variation criteria) were not chosen empirically by optimizing classification performance, but were instead derived from the expected dynamical signatures of the corresponding bifurcations. For example, thresholds associated with spike-amplitude reduction or baseline shifts were introduced to capture the characteristic qualitative behavior predicted by bifurcation theory rather than to maximize statistical accuracy on a labeled dataset.

For this reason, we believe that performance metrics such as confusion matrices, precision, and recall are not the most appropriate validation tools for the proposed algorithm. Our classifier is not intended as a supervised machine-learning method but rather as a deterministic implementation of theoretical bifurcation criteria that can be applied directly to membrane voltage recordings. Nevertheless, following your suggestion, we expanded the Methods section and included additional examples in the Supplementary Material (S3 Fig. and S4 Fig.) to illustrate the behavior of the classifier. We also discuss the sensitivity of these thresholds and explain why moderate variations do not substantially alter the classification outcome.

Following your suggestion we computed a 2D plot about degeneracy group at fixed potassium concentration. In particular we decided to project all the point in the gNa-gCl space and we color the point according to the number of points in the hidden variable. The figure have been added in Supplementary Material (S1 Fig.).

We further clarified that some bursting classes are intentionally grouped together when their underlying bifurcations cannot be unambiguously distinguished from membrane voltage traces alone. This limitation does not arise from the deterministic rules themselves but from the intrinsic lack of information contained in a single observable. Distinguishing such cases would require access to additional dynamical variables or a full phase-space reconstruction. We have explicitly discussed this limitation in the revised manuscript.

Following your suggestion, we also expanded the discussion of the classifier limitations and the relationship between classification uncertainty and the boundaries separating different dynamical regions. In particular, we now discuss that transitions occurring near the borders of degeneracy regions may reflect either genuine changes in the underlying bifurcation structure or the intrinsic ambiguity associated with voltage-only classification.

Finally, concerning the definition of neuronal resilience, our objective was to quantify how much increase in extracellular potassium a neuron can tolerate, given its intrinsic conductance configuration, before undergoing a transition to a different dynamical regime. Since elevated extracellular potassium is a well-established hallmark of pathological conditions such as epileptic seizures, we considered the first transition point to be the most meaningful measure of resilience. Tracking the entire potassium interval over which a given activity pattern persists is certainly an interesting complementary characterization, but it addresses a different question, namely the stability range of an already established regime rather than the neuron's resistance to the onset of pathological dynamics. We have clarified this motivation in the revised manuscript. To conclude , extending the model to include additional ionic mechanisms could be particularly informative when investigating degeneracy in phenomena involving other ionic dynamics, such as persistent Na^+ or Ca^{2+} currents, variations in the leak reversal potential, or dynamic intracellular chloride regulation. In particular, given the prominent role of chloride in shaping resilience in our results, a valuable future extension would be to incorporate synaptic inputs and their associated chloride dynamics. Such an extension would allow us to assess whether the conclusions obtained with the present reduced model generalize to more biophysically detailed and physiologically realistic conditions.

Minor Comments:

The unusually high spike frequency results from the timescales chosen for the fast subsystem. A more physiologically realistic frequency could be obtained by rescaling the time constants of the two fast equations while keeping their ratio unchanged. This rescaling would preserve the bifurcation structure and, therefore, would not affect the qualitative results. We did not apply it because matching a physiologically realistic spike frequency was not necessary for the qualitative analysis performed in this study.

All the other minor comments have been addressed in the revised manuscript.
