# Supplementary material for: Degeneracy in neuronal bifurcation landscapes: Equivalent bifurcation sequences across distinct parameter sets
Source: PLoS Comput Biol. 2026 Oct 30;22(10):e1014893. doi: 10.1371/journal.pcbi.1014893 (PMC13739234; doi:10.1371/journal.pcbi.1014893)
Supplement: Peer review history

# Peer review: Decision Letter 0

**DOI:** 10.1371/journal.pcbi.1014893.r001

**Authors:** Lyle Graham, Waldo Nogueira

15 Apr 2026

Degeneracy in neuronal bifurcation landscapes: equivalent bifurcation sequences across distinct parameter sets

PLOS Computational Biology

Dear Dr. Cafaro,

Thank you for submitting your manuscript to PLOS Computational Biology. After careful consideration, we feel that it has merit but does not fully meet PLOS Computational Biology's publication criteria as it currently stands. Therefore, we invite you to submit a revised version of the manuscript that addresses the points raised during the review process.

Please submit your revised manuscript by Jun 15 2026 11:59PM. If you will need more time than this to complete your revisions, please reply to this message or contact the journal office at ploscompbiol@plos.org. When you're ready to submit your revision, log on to https://www.editorialmanager.com/pcompbiol/ and select the 'Submissions Needing Revision' folder to locate your manuscript file.

Please include the following items when submitting your revised manuscript:

* A letter that responds to each point raised by the editor and reviewer(s). You should upload this letter as a separate file labeled 'Response to Reviewers'. This file does not need to include responses to formatting updates and technical items listed in the 'Journal Requirements' section below.

* A marked-up copy of your manuscript that highlights changes made to the original version. You should upload this as a separate file labeled 'Revised Manuscript with Track Changes'.

* An unmarked version of your revised paper without tracked changes. You should upload this as a separate file labeled 'Manuscript'.

If you would like to make changes to your financial disclosure, competing interests statement, or data availability statement, please make these updates within the submission form at the time of resubmission. Guidelines for resubmitting your figure files are available below the reviewer comments at the end of this letter

We look forward to receiving your revised manuscript.

Kind regards,

Waldo Nogueira

Academic Editor

PLOS Computational Biology

Lyle Graham

Section Editor

PLOS Computational Biology

**Journal Requirements:**

1) Please ensure that the CRediT author contributions listed for every co-author are completed accurately and in full.

At this stage, the following Authors/Authors require contributions: Gianmarco Cafaro. Please ensure that the full contributions of each author are acknowledged in the "Add/Edit/Remove Authors" section of our submission form.

The list of CRediT author contributions may be found here: https://journals.plos.org/ploscompbiol/s/authorship#loc-author-contributions

2) We ask that a manuscript source file is provided at Revision. Please upload your manuscript file as a .doc, .docx, .rtf or .tex. If you are providing a .tex file, please upload it under the item type u2018LaTeX Source Fileu2019 and leave your .pdf version as the item type u2018Manuscriptu2019.

3) Please upload all main figures as separate Figure files in .tif or .eps format. For more information about how to convert and format your figure files please see our guidelines:

https://journals.plos.org/ploscompbiol/s/figures

4) We note that your Data Availability Statement is currently as follows: "All relevant data are within the manuscript and its Supporting Information files." However, you have not uploaded any supporting information files. Please confirm at this time whether or not your submission contains all raw data required to replicate the results of your study. Authors must share the “minimal data set” for their submission. PLOS defines the minimal data set to consist of the data required to replicate all study findings reported in the article, as well as related metadata and methods (https://journals.plos.org/plosone/s/data-availability#loc-minimal-data-set-definition).

For example, authors should submit the following data:

1) The values behind the means, standard deviations and other measures reported;

2) The values used to build graphs;

3) The points extracted from images for analysis..

Authors do not need to submit their entire data set if only a portion of the data was used in the reported study.

If your submission does not contain these data, please either upload them as Supporting Information files or deposit them to a stable, public repository and provide us with the relevant URLs, DOIs, or accession numbers. For a list of recommended repositories, please see https://journals.plos.org/plosone/s/recommended-repositories.

If there are ethical or legal restrictions on sharing a de-identified data set, please explain them in detail (e.g., data contain potentially sensitive information, data are owned by a third-party organization, etc.) and who has imposed them (e.g., an ethics committee). Please also provide contact information for a data access committee, ethics committee, or other institutional body to which data requests may be sent. If data are owned by a third party, please indicate how others may request data access.

5) Please provide a completed 'Competing Interests' statement, including any COIs declared by your co-authors. If you have no competing interests to declare, please state "The authors have declared that no competing interests exist". Otherwise please declare all competing interests beginning with the statement "I have read the journal's policy and the authors of this manuscript have the following competing interests:"".

Note: If the reviewer comments include a recommendation to cite specific previously published works, please review and evaluate these publications to determine whether they are relevant and should be cited. There is no requirement to cite these works unless the editor has indicated otherwise.

**Reviewers' comments:**

Reviewer's Responses to Questions

Reviewer #1: The authors investigate the firing patterns of a single-compartment neuron model with three conductances (Na, Cl, Ca) under systematic variation of conductances and extracellular potassium concentration. Elevated extracellular potassium concentration, as observed during epileptic seizures, can induce bursting activity characterized by specific onset and offset bifurcations. The authors classify bifurcation sequences solely from membrane voltage time series. This voltage-only approach is novel, as classical methods rely on additional dynamical variables, and important since experiments typically provide only membrane voltage recordings. Their analysis further identifies regions of degeneracy in which distinct parameterizations produce identical dynamical behavior. They introduce resilience as the minimal extracellular potassium concentration required to induce a given activity pattern and potassium conductance having the largest effect on it.

The manuscript accurately and fairly summarizes prior work, identifies a clear gap, and formulates a well-defined research question that it addresses with a significant contribution. The methods are appropriate and described in sufficient detail to allow reproduction. Given the focused research question, the statistical analysis is adequate and conceptually sound, and the work appears technically correct. Broader generalization would require additional statistics across more initializations and parameterizations. Assumptions are justified and the results support the conclusions without overinterpretation. The discussion places the findings in context and lists limitations. The abstract reflects the content faithfully.

The presentation and language are clear. Figures and tables are generally accurate, clear, and necessary. A particular strength of the study is its logical design and the development of a practical classification tool that may benefit experimental research.

The manuscript is suitable for PLOS Computational Biology, as it advances understanding of epileptic activity through a controlled biophysical model and a novel computational classification approach. The minor revisions suggested below would further improve the manuscript.

Content

* l. 205 Table 1: Why does the number of sampled points differ between parameters? How were the lower and upper bounds of each parameter range determined?

* l. 296 Please mark the nine points in Fig. 4 that are analyzed in detail in Fig. 5.

* l. 600-601 What does the vertical bar in SN-SupH | SupH-SH and SN-SupH | SupH-Infinity denote? Please explain this when introducing the bursting categories.

* l. 598-599 What do "type 1" and "type 2" refer to? Please define these categories at first mention.

Clarity

* Consider summarizing all bifurcation types (abbreviation, full name, key features) in a single table and referring to them thereafter by abbreviation only.

* Please reference figures in order of appearance and place each figure after its first mention (e.g., consider switching the left and right panels of Figs. 2 and 3).

* Consider numbering all displayed equations, as this may facilitate referencing in future work / discussions.

* l. 137-146 Consider referring to Eq. 1a-d already here.

* l. 164-174 An explicit reference to the algorithm in Section 5.3 would be helpful.

* l. 187 An explicit reference to Section 5.2 would be helpful.

* l. 189 Refer to Section 5.3 instead of Section 5.

* l. 195 Refer to Section 5.2 instead of "Materials and Methods."

* l. 224 (Fig. 1) The color bar for K_bath is unnecessary given the two fixed values. Consider labeling the three configurations as (a), (b), (c) and the 3D plot as (d), marking the points directly rather than using arrows. Arranging subplots in columns would avoid repetition of current traces.

* l. 256 In Fig. 2, mark the three points in conductance space corresponding to the membrane traces.

* l. 256, 261 Consider adding a legend to the 3D plots explaining the color coding.

* l. 256, 261 Consider merging Figs. 2 and 3 or separating their discussion more systematically in the text.

* l. 256, 261 Use consistent colors for membrane traces and their corresponding points in conductance space.

* l. 352 In Fig. 6, use subfigure labels (a), (b), (c), (d) instead of a.1, a.2, b1, b2.

* l. 352 Define the abbreviation "DB" earlier or omit since it is used only once.

* l. 547 Table 3 may be placed earlier, as it defines variables in Eq. 1.

* l. 547 Please specify the unit (ms) for the time constant.

* l. 573 Consider a more specific title than "Deterministic classifier algorithm," indicating that it classifies bifurcation types.

* l. 573 Section 5.3 would benefit from a structured algorithm listing with numbered steps and formulas.

Formatting

* l. 205 Table 1 (and elsewhere, including figure labels) Typeset Na, Cl, K, and bath upright in math expressions.

* l. 223 Ensure consistent spacing in figure references (e.g., "Fig. 1").

* l. 224 (caption) "pA" appears to have formatting issues; please correct.

* l. 224 Fig. 1 Label the y-axis as V_m for consistency.

* l. 508 Typeset the differential operator d upright.

Typos

* l. 115 corresponds -> correspond

* l. 224 (Fig. 1 caption) fist -> first

* l. 427 model -> models

* l. 545 conducatance -> conductance

Reviewer #2: General assessment

The authors address a topic of general interest: Are regions of similar

spiking behaviour compact sets in parameter space and how are these

region influenced by other parameters. This leads to a concrete concept

of resilience for spiking neurons. Their study relies on the analysis of

a conductance-based model with potassium concentration dynamics. Some of

the concrete findings of the article are relatively intuitive and not

entirely new and it might be necessary to add some content before the

manuscript is suitable for publication.

Comments

The reviewer appreciates the concrete definition of resilience in

section 3.4. as the minimum extracellular potassium concentration that

elicits a pattern. The gradient of this resilience increase is plotted

as vectors for all conductance combinations. This, since not trivial to

read, e.g. in Fig. 6, might benefit from additional explanations and

visualisation. This is particularly severe, since some of the

conclusions are not easily seen in the plots. Contrary to the text (ll.

346), it seems that for high starting K_(Bath) the g_(K) conductance

suddenly has smaller contribution. It is difficult to read this plot if

the conductance-axis are scaled differently. This alludes to the more

general discussion of how to compare changes in conductances as

different as leak, potassium or sodium. The change to polar coordinates

does not improve the readability either. This should be improved on.

It might be prudent not to over generalise from simulations with one (to

some extend idiosyncratic) model. The statement in ll. 364 “The g_(K)

component is consistently positive and larger than the others,

indicating that increasing potassium conductance strengthens the

neuron’s resistance to transitions in activity patterns.” The authors

show only resistance to potassium concentration perturbations, hence it

is almost trivially so that the potassium conductance has the strongest

influence. The authors discuss this point in ll. 381 onwards. They make

a statement about sodium (due to the inverse relation between sodium and

potassium, probably referring to equations 4a-g), but show no data. But

it would important for the article, to also analyse perturbations in

intracellular sodium concentrations or chloride. Is it then still g_(K)

or actually g_(Na) that influences resilience. In particular, relations

4a-g rely on the electro-neutrality assumption. Bulk electro-neutrality

is locally disobeyed during AP dynamics and in general only eliminates

on of the concentration degrees of freedom, see (Hübel and Dahlem 2014)

for a more extensive discussion. Considering the importance of the

issues for the submitted manuscript it seems necessary to expand on

these matters and include a more extensive analysis of sodium currents.

In other words, the consequences of the particular choice of model are

unclear and not presented. In particular, the model simplification. For

example the instantaneous sodium dynamics, the slaving of the sodium

inactivation to the potassium gate are all not really necessary and not

motivated.

I have some mild criticism on how the present work in embedded into the

existing literature. Some of the statements presented as findings here,

are known in older articles. For example in ll. 268 the authors state

“our results show that both intrinsic neuronal properties and external

ionic concentration jointly determine the activity patterns exhibited by

the model”. This, to some extend trivial insight in a non-linear system,

has been reported on extensively both in the modelling literature

Behbood et al. (2024) as well as in experimental work (Contreras et al.

2021) beyond the work of Eve Marder. The authors could strengthen their

manuscript by referring and discussing these older studies.

The semi-manual classification algorithm of onset bifurcation described

in the method section, might be exemplified in the results section,

showing the reader how it works and providing some statistics on its

performance.

Detailed remarks

Please name and introduce all state variables and parameters in the

Materials and Methods section 5.1, e.g. what is K_(g).

References

Behbood, Mahraz, Louisiane Lemaire, Jan-Hendrik Schleimer, and Susanne

Schreiber. 2024. “The Na+/k+-ATPase Generically Enables Deterministic

Bursting in Class i Neurons by Shearing the Spike-Onset Bifurcation

Structure.” PLOS Computational Biology 20 (August): e1011751.

https://doi.org/10.1371/journal.pcbi.1011751.

Contreras, Susana Andrea, Jan-Hendrik Schleimer, Allan T. Gulledge, and

Susanne Schreiber. 2021. “Activity-Mediated Accumulation of Potassium

Induces a Switch in Firing Pattern and Neuronal Excitability Type.” PLOS

Computational Biology 17 (5): e1008510.

https://doi.org/10.1371/journal.pcbi.1008510.

Hübel, Niklas, and Markus A. Dahlem. 2014. “Dynamics from Seconds to

Hours in Hodgkin-Huxley Model with Time-Dependent Ion Concentrations and

Buffer Reservoirs.” PLOS Computational Biology 10 (12): e1003941.

https://doi.org/10.1371/journal.pcbi.1003941.

Hübel, Niklas, Eckehard Schöll, and Markus A. Dahlem. 2014. “Bistable

Dynamics Underlying Excitability of Ion Homeostasis in Neuron Models.”

PLOS Computational Biology 10 (5): e1003551.

https://doi.org/10.1371/journal.pcbi.1003551.

Reviewer #3: The manuscript proposes an interesting computational study of “degeneracy” at the level of dynamical transitions in single-neuron models. Using a four-variable slow/fast model with three tunable maximal conductances (gNa, gK, gCl) and an external potassium drive, the authors show that (i) several distinct conductance triplets can elicit the same onset/offset bifurcation sequence and that (ii) these “degeneracy regions’’ rearrange when the potassium is varied and also that (iii) the minimal potassium needed to trigger a given pattern (“resilience”) depends most strongly on gK. They also describe a pragmatic voltage-only classifier of bifurcation types that could, in principle, be used on experimental traces.

The study is timely, touches on a recurring question in computational neuroscience, and is generally well written. The code is also provided (GitHub is mentioned, and the repository is populated). Nevertheless, it is my view that several conceptual, methodological, and presentation issues should be addressed before the work can be recommended for publication.

Comments:

1) Section 5.3 The deterministic rules (baseline jump, amplitude ratio <0.8, 5000-spike threshold, etc.) are introduced ad-hoc. Please supply a quantitative validation: confusion matrix, precision/recall. A small discussion of the robustness to additive noise would be interesting.

2) How sensitive are the thresholds (0.8)? A short sensitivity analysis, or at least a justification based on theoretical expectations, is needed.

3) It is my understanding that some classes are collapsed (e.g., SN-SupH|SupH-SH) when they cannot be separated by the rules. Is there evidence that they are truly inseparable from Vm only? If not, could a simple supervised classifier (e.g. decision tree) do better?

4) The manuscript alternates between “pattern class’’ and “degeneracy region’’ but the relationship remains confusing. A degeneracy region is first defined at a fixed potassium bath, yet later the term seems to migrate with the potassium bath. Consider providing a formal definition in the Methods.

5) A 2-D projection (e.g., gNa vs gK with gCl coded by color) showing one complete degeneracy region (or what people call a ‘level set’), to convince the reader that it is indeed connected and compact. It could go with one of the 3D plots the authors propose.

6) A short discussion on whether the borders of such regions correspond to codimension-2 bifurcations or to a failure of the classifier would also be interesting.

7) Why is resilience defined only as the minimal potassium bath at which the pattern first appears? Would it not be more informative to track the entire interval of the potassium bath over which the pattern persists?

8) Only three maximal conductances are varied; all time constants, reversal potentials, and pump parameters are frozen. Some discussion of how the conclusions might (or might not) extend to a more biophysically detailed model with, for example, persistent Na+, Ca2+, leak reversal, or dynamic chloride would be welcome.

Minor comments

Citation style oscillates between numeric and author-year. Plos CB uses only numerical

Fig. 1 legend: “fist parameter sets” - first.

The threshold “5000 spikes” looks suspiciously high. Are you sure this is correct?

Spherical coordinates: please check limits, it might be that theta is [0,pi] and not [0, 2pi]

**********

**Have the authors made all data and (if applicable) computational code underlying the findings in their manuscript fully available?**

The PLOS Data policy requires authors to make all data and code underlying the findings described in their manuscript fully available without restriction, with rare exception (please refer to the Data Availability Statement in the manuscript PDF file). The data and code should be provided as part of the manuscript or its supporting information, or deposited to a public repository. For example, in addition to summary statistics, the data points behind means, medians and variance measures should be available. If there are restrictions on publicly sharing data or code —e.g. participant privacy or use of data from a third party—those must be specified.

Reviewer #1: **No:**  The code is provided via GitHub (https://github.com/GianmarCafaro/Pattern_Detection/blob/main/widget_classifier.ipynb), and the authors state that all relevant data are included in the manuscript and Supporting Information. However, the simulated raw data do not appear to be available in the repository, and the Supporting Information files are either not accessible in the review system or not provided. This should be clarified.

Reviewer #2: Yes

Reviewer #3: Yes

**********

PLOS authors have the option to publish the peer review history of their article (what does this mean?). If published, this will include your full peer review and any attached files.

If you choose “no”, your identity will remain anonymous but your review may still be made public.

**Do you want your identity to be public for this peer review?**  For information about this choice, including consent withdrawal, please see our Privacy Policy.

Reviewer #1: No

Reviewer #2: No

Reviewer #3: No

[NOTE: If reviewer comments were submitted as an attachment file, they will be attached to this email and accessible via the submission site. Please log into your account, locate the manuscript record, and check for the action link "View Attachments". If this link does not appear, there are no attachment files.]

**Figure resubmission:**

After uploading your figures to PLOS’s NAAS tool - https://ngplosjournals.pagemajik.ai/artanalysis, NAAS will process the files provided and display the results in the "Uploaded Files" section of the page as the processing is complete. If the uploaded figures meet our requirements (or NAAS is able to fix the files to meet our requirements), the figure will be marked as "fixed" above. If NAAS is unable to fix the files, a red "failed" label will appear above. When NAAS has confirmed that the figure files meet our requirements, please download the file via the download option, and include these NAAS processed figure files when submitting your revised manuscript.

**Reproducibility:**

To enhance the reproducibility of your results, we recommend that authors of applicable studies deposit laboratory protocols in protocols.io, where a protocol can be assigned its own identifier (DOI) such that it can be cited independently in the future. Additionally, PLOS ONE offers an option to publish peer-reviewed clinical study protocols. Read more information on sharing protocols at https://plos.org/protocols?utm_medium=editorial-email&utm_source=authorletters&utm_campaign=protocols

# Author comment: Author response to Decision Letter 1

**DOI:** 10.1371/journal.pcbi.1014893.r002

13 Aug 2026

Attachment Submitted filename: Response to Reviewers.pdf

# Peer review: Decision Letter 1

**DOI:** 10.1371/journal.pcbi.1014893.r003

**Authors:** Lyle Graham, Waldo Nogueira

12 Oct 2026

Dear Dr. Cafaro,

We are pleased to inform you that your manuscript 'Degeneracy in neuronal bifurcation landscapes: equivalent bifurcation sequences across distinct parameter sets' has been provisionally accepted for publication in PLOS Computational Biology.

Before your manuscript can be formally accepted you will need to complete some formatting changes, which you will receive in a follow up email. A member of our team will be in touch with a set of requests.

Please note that your manuscript will not be scheduled for publication until you have made the required changes, so a swift response is appreciated.

IMPORTANT: The editorial review process is now complete. PLOS will only permit corrections to spelling, formatting or significant scientific errors from this point onwards. Requests for major changes, or any which affect the scientific understanding of your work, will cause delays to the publication date of your manuscript.

Should you, your institution's press office or the journal office choose to press release your paper, you will automatically be opted out of early publication. We ask that you notify us now if you or your institution is planning to press release the article. All press must be co-ordinated with PLOS.

Thank you again for supporting Open Access publishing; we are looking forward to publishing your work in PLOS Computational Biology.

Best regards,

Waldo Nogueira

Academic Editor

PLOS Computational Biology

Lyle Graham

Section Editor

PLOS Computational Biology

***********************************************************

Reviewer's Responses to Questions

**Comments to the Authors:**

**Please note here if the review is uploaded as an attachment.**

Reviewer #1: Thank you for your revision. All my major comments have been addressed and I have no further comments.

Reviewer #2: Thank you very much for the continued on the manuscript. The presentation is now substantially easier to follow and the scientific findings a are motivated. In particular, highlighting the role of the, in the modeling literature often underrepresented chloride channels is much appreciated.

The biophysical interpretation of the robustness measure adds an intuitive explanations to the findings and corroborates the numerical studies.

It seems a fair point to restrict the analysis to a particular learning tasks, after all, one has to start somewhere. And the goal of the article was not to outperform cutting edge machine learning but to gain biophysical insight. In that sense, the choice to make the model assumptions more explicit and reduce the generality claims a bit is a meaning full compromise.

I would be happy to see this article published and recommend it to colleagues.

Reviewer #3: The authors addressed all my questions and I believe the manuscript has improved. I have no more concerns.

**********

**Have the authors made all data and (if applicable) computational code underlying the findings in their manuscript fully available?**

The PLOS Data policy requires authors to make all data and code underlying the findings described in their manuscript fully available without restriction, with rare exception (please refer to the Data Availability Statement in the manuscript PDF file). The data and code should be provided as part of the manuscript or its supporting information, or deposited to a public repository. For example, in addition to summary statistics, the data points behind means, medians and variance measures should be available. If there are restrictions on publicly sharing data or code —e.g. participant privacy or use of data from a third party—those must be specified.

Reviewer #1: None

Reviewer #2: Yes

Reviewer #3: Yes

**********

PLOS authors have the option to publish the peer review history of their article (what does this mean?). If published, this will include your full peer review and any attached files.

If you choose “no”, your identity will remain anonymous but your review may still be made public.

**Do you want your identity to be public for this peer review?**  For information about this choice, including consent withdrawal, please see our Privacy Policy.

Reviewer #1: No

Reviewer #2: **Yes:**  Jan-Hendrik Schleimer

Reviewer #3: No

# Editor report: Acceptance letter

**DOI:** 10.1371/journal.pcbi.1014893.r004

**Authors:** Lyle Graham, Waldo Nogueira

PCOMPBIOL-D-25-02268R1

Degeneracy in neuronal bifurcation landscapes: equivalent bifurcation sequences across distinct parameter sets

Dear Dr Cafaro,

I am pleased to inform you that your manuscript has been formally accepted for publication in PLOS Computational Biology. Your manuscript is now with our production department and you will be notified of the publication date in due course.

The corresponding author will soon be receiving a typeset proof for review, to ensure errors have not been introduced during production. Please review the PDF proof of your manuscript carefully, as this is the last chance to correct any errors. Please note that major changes, or those which affect the scientific understanding of the work, will likely cause delays to the publication date of your manuscript.

Soon after your final files are uploaded, unless you have opted out, the early version of your manuscript will be published online. The date of the early version will be your article's publication date. The final article will be published to the same URL, and all versions of the paper will be accessible to readers.

For Research, Software, and Methods articles, you will receive an invoice from PLOS for your publication fee after your manuscript has reached the completed accept phase. If you receive an email requesting payment before acceptance or for any other service, this may be a phishing scheme. Learn how to identify phishing emails and protect your accounts at https://explore.plos.org/phishing.

Thank you again for supporting PLOS Computational Biology and open-access publishing. We are looking forward to publishing your work!

With kind regards,

Janani Seenivasan

PLOS Computational Biology | Carlyle House, Carlyle Road, Cambridge CB4 3DN | United Kingdom ploscompbiol@plos.org | Phone +44 (0) 1223-442824 | ploscompbiol.org | @PLOSCompBiol
